# Pharmacological and pupillary evidence for the noradrenergic contribution to reinforcement learning in Parkinson’s disease

**DOI:** 10.1038/s42003-025-08627-2

**Published:** 2025-08-14

**Authors:** Claire O’Callaghan, Frank H. Hezemans, Naresh Subramaniam, Rong Ye, Kamen A. Tsvetanov, Alexander G. Murley, Negin Holland, Isabella F. Orlando, Ralf Regenthal, Roger A. Barker, Caroline H. Williams-Gray, Luca Passamonti, Trevor W. Robbins, James B. Rowe

**Affiliations:** 1 Brain and Mind Centre and School of Medical Sciences, Faculty of Medicine and Health, University of Sydney, Sydney, Australia; 2 MRC Cognition and Brain Sciences Unit, University of Cambridge, Cambridge, UK; 3 Department of Clinical Neurosciences and Cambridge University Hospitals NHS Trust, University of Cambridge, Cambridge, UK; 4 Department of Psychiatry and Psychotherapy, University Hospital and Faculty of Medicine, Eberhard Karls University of Tübingen, Tübingen, Germany; 5 Department of Psychiatry, University of Cambridge, Cambridge, UK; 6 Department of Psychology, University of Cambridge, Cambridge, UK; 7 Division of Clinical Pharmacology, Rudolf-Boehm-Institute for Pharmacology and Toxicology, University of Leipzig, Leipzig, Germany; 8 John van Geest Centre for Brain Repair, Department of Clinical Neurosciences, University of Cambridge, Cambridge, Cambridge, UK; 9 Wellcome Trust - Medical Research Council Stem Cell Institute, University of Cambridge, Cambridge, Cambridge, UK; 10 Behavioural and Clinical Neuroscience Institute, University of Cambridge, Cambridge, UK

**Keywords:** Cognitive neuroscience, Parkinson's disease

## Abstract

Noradrenaline plays an integral role in learning by optimising behavioural strategies and facilitating choice execution. Testing the noradrenergic framework of learning in the context of human diseases offers a test bed for current normative neuroscience theories and may also indicate therapeutic potential. Parkinson’s disease is often considered as a model of dopamine deficits, including dopamine’s role in reinforcement learning. However, noradrenergic function is also severely impaired by Parkinson’s disease, contributing to cognitive deficits. Using a single dose of the noradrenaline reuptake inhibitor atomoxetine in people with Parkinson’s disease (in a randomised double-blind placebo-controlled crossover design), we show improvements in learning compared to placebo. Computational cognitive modelling confirmed a substantial shift in the decision noise parameter, indicative of more exploitative choices. This response pattern closely resembled that of age-matched controls and simulations of optimal response strategies. Pupillometry revealed increased baseline pupil diameter under atomoxetine, which correlated with behavioural improvements, and a heightened phasic pupillary response to feedback. Our findings confirm the noradrenergic contribution to reinforcement learning, and in doing so they challenge the simple interpretation of tonic-phasic locus coeruleus firing patterns based on pupillometry. Noradrenergic modulation is a potential treatment strategy for cognitive symptoms in Parkinson’s disease and related disorders.

## Abstract (web summary)

Using pharmacology, pupillometry and cognitive modelling, evidence for a noradrenergic contribution to reinforcement learning is shown in Parkinson’s disease.

## Introduction

Neuromodulators provide dynamic support for the optimal balance of flexibility and stability needed for adaptive behaviour. Here we focus on the role of noradrenaline with its diverse influences on cognitive processes^1^ and vulnerability in neurodegenerative disease^2,3^. The brain’s main source of noradrenaline is the pontine locus coeruleus, in which neuronal activity is associated with critical phases of learned behaviour. These include initial stimulus encoding, processing feedback and executing choices^4^. Diffuse locus coeruleus-noradrenergic projections modulate both the stable expression of learned behaviour and the flexibility required to learn new behaviours when environmental contingencies change.

Phasic locus coeruleus activity is time-locked to salient sensory stimuli^5^. While this is a driver of arousal-mediated learning, a closer association is observed between phasic firing and the behavioural response^6–9^. This suggests that locus coeruleus activity signals brain-wide decisional processes, and in turn facilitates the influence of those decisional processes on subsequent behaviour^10^. For this reason, noradrenaline has been hypothesised to control inverse temperature in reinforcement learning^11^—a parameter capturing the balance between flexible exploration of actions to support new learning vs. stable expression of learned actions. When environmental contingencies change, the mismatch between an action and its anticipated outcome is accompanied by robust locus coeruleus responding and cerulo-cortical noradrenaline release in prefrontal cortex^12,13^. Indeed, locus coeruleus stimulation increases behavioural variability during value-based decision making^14^, and accelerates correct responding to a changed reward contingency^15^ or to a new rule^16^. Pharmacological manipulations and pupillary recordings in humans also implicate locus coeruleus-noradrenergic activity in learning under uncertain, volatile conditions^17–19^.

An overarching function of noradrenaline in learning is therefore to drive plasticity and reconfigure behavioural strategies in the face of novel, unexpected events or changes in reinforcement contingency^13,20–23^. This role directly relates to locus coeruleus firing patterns and functional anatomy. For example, selective responding to task-relevant stimuli occurs under intermediate tonic levels with phasic firing, with response selectivity decreasing at higher tonic levels^24^. Intermediate levels permit stability while a behaviour remains useful, while higher levels permit disengagement so as to sample alternate behavioural options when a new strategy may be better. Widespread cortical projections from and to the locus coeruleus make it well placed to integrate information from across the brain and, in turn, broadcast a global signal to bias subsequent learning and behaviour^25^. Noradrenergic actions at target sites include modulation of the gain (responsivity) of circuits^24^, with different adrenergic receptor subtypes strengthening vs. weakening functional circuits to maintain or reconfigure behaviour^20,26,27^.

The locus coeruleus is one of the earliest sites of pathology in Parkinson’s disease^28^, and is associated with diverse cognitive and neuropsychiatric symptoms^3^. The functional significance of this noradrenergic deficit complements the better-known role of striatal dopamine in cognition, and in reinforcement learning in particular^29^. The effects of Parkinson’s disease and dopaminergic medication have differentiated the function of phasic bursts activating low-affinity striatal D1 receptors from phasic dips activating high-affinity D2 receptors, which in turn drive positive and negative reinforcement learning, respectively^30–32^. What then is the role of phasic and tonic firing of cerulo-cortical projections? A noradrenergic role in reinforcement learning would be important to improve the disease model and explain learning deficits in Parkinson’s disease, as well as open new therapeutic options^2^.

A noradrenergic therapeutic strategy of special interest is the inhibition of noradrenaline reuptake. This strategy mirrors serotonergic reuptake inhibition in depression and anxiety, dopamine reuptake inhibition in Parkinson’s disease and GABA reuptake inhibition in epilepsy. The noradrenergic reuptake inhibitor atomoxetine increases extracellular levels of noradrenaline and phasic-to-tonic locus coeruleus firing^33^. The drug may also increase extracellular dopamine in the frontal cortex, although not in the striatum^34^, where there is a paucity of noradrenergic projections^35^. Therefore, atomoxetine permits a manipulation of noradrenaline that is unlikely to directly influence the striatal dopaminergic pathways implicated in Parkinson’s disease reinforcement learning.

Here, we assess the noradrenergic contribution to reinforcement learning in the context of Parkinson’s disease, using a single dose of the noradrenergic reuptake inhibitor atomoxetine in a double-blind, cross-over, placebo-controlled design. Principal analyses focus on the within-Parkinson’s crossover design, with normative data from age-matched controls also provided, for context. By combining the task with pupillometry, proposed as a proxy for locus coeruleus activity^36^, we test the hypothesis that noradrenaline supports reinforcement learning.

## Results

### Demographics and clinical assessments

Patient and control groups were matched for demographics, but as expected, people with Parkinson’s disease had a lower ACE-R total score (see Table 1).

**Table 1:** Demographics and clinical assessments of participants in their regular medication state

	PD	Control	*p-value*
Age (years)	67.11 (7.05) *[50–80]*	65.35 (5.32) *[53–78]*	0.368
Education (years)	14.05 (2.27) *[10–18]*	14.65 (3.10) *[8–20]*	0.457
Male/Female	15 4	15 / 11	0.240
MMSE	29.47 (0.70) *[28–30]*	29.77 (0.51) *[28–30]*	0.128
MoCA	28.11 (1.76) *[24–30]*	28.58 (1.39) *[25–30]*	0.340
ACE-R	94.89 (3.71) *[84–100]*	97.58 (3.16) *[84–100]*	**0.015**
MDS-UPDRS-III	28.42 (11.60) *[6–55]*		
Hoehn and Yahr stage	2.26 (0.45) *[2–3]*		
Disease duration (years)	4.15 (1.72) *[1.6–8]*		
Levodopa equivalent daily dose (mg/day)	644.55 (492.81) *[80–1984]*		

Data are presented as mean (SD), *[range]*. Comparisons of patient and control groups were performed with independent samples *t*-tests or contingency tables as appropriate. Bold denotes significant at *p* < 0.05.

*MMSE* mini-mental state examination, *MoCA* Montreal cognitive assessment, *ACE-R* revised Addenbrooke’s cognitive examination, *MDS-UPDRS* Movement Disorders Society unified Parkinson’s disease rating scale.

### Reinforcement learning task performance

**Fig. 1:**
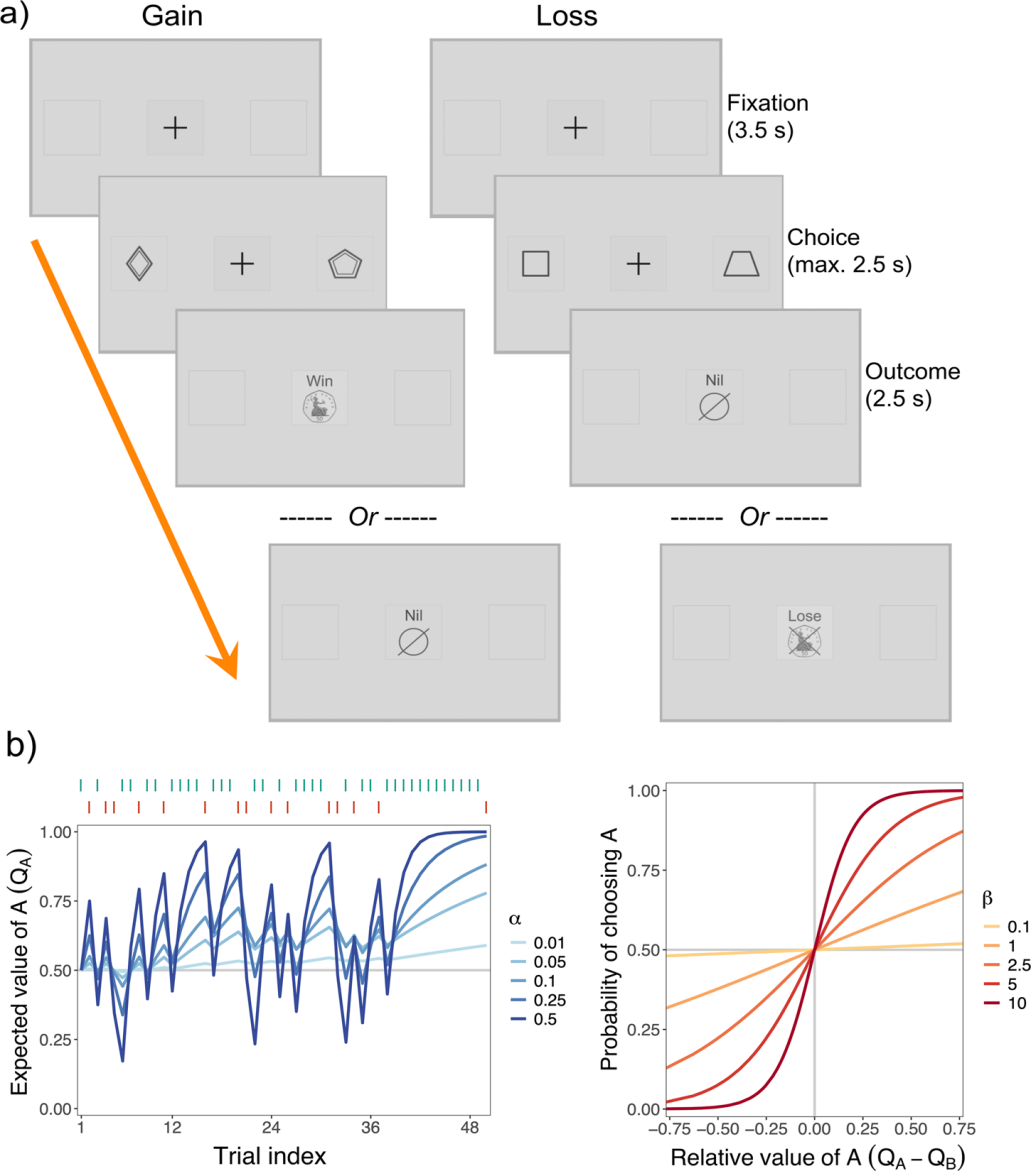
Trial structure of the reinforcement learning task and Q-learning model. **a** *Trial structure*: The task involved gain and loss trials, where choosing the correct stimulus resulted in a win of 50 pence versus getting “nil” (gain) or where choosing the correct stimulus resulted in getting “nil” versus losing 50 pence (loss). **b** *Q-learning model*: Left panel (learning rate, $\alpha$) shows trajectories of the expected value of a given stimulus-action pair for an example time series of outcomes (green and red dashes), under a range of values of the learning rate; Right panel (inverse temperature, $\beta$) shows the probability of performing a given action as a function of the expected value of that action relative to the expected value of an alternative action, under a range of values of the inverse temperature.

Participants performed a reinforcement learning task where two pairs of visual stimuli were probabilistically associated with monetary gains or losses. One pair (the gain condition) was associated with winning 50 pence or getting nothing; the other pair (the loss condition) was associated with losing 50 pence or getting nothing. In each pair, the correct choice (i.e., selecting the winning stimulus in gain, or avoiding the losing stimulus in loss) was probabilistically rewarded 75% of the time (see the “Methods” section; Fig. 1a). Performance was first analysed behaviourally, prior to applying a *Q*-learning model.

**Fig. 2:**
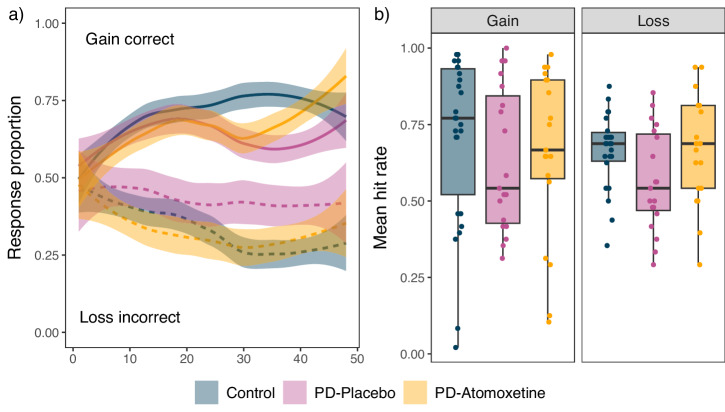
Reinforcement learning task performance. **a** *Learning over trials*: Smoothed learning curve across the 48 trials (*x*-axis). Proportion of choices where the winning stimulus was chosen in the gain condition (upper graph; solid line) and where the losing stimulus was avoided in the loss condition (lower graph; dashed line); ribbon represents standard error. **b** *Overall accuracy*: Average hit rate, i.e., the proportion of trials where the correct (reward-maximising) stimulus was chosen, plotted separately for each task condition and group/drug condition. Individual participants are represented by dots (*n* = 19 PD-atomoxetine/PD-placebo; *n* = 26 controls). Box plot elements: centre line, median; box limits, first and third quartiles; whiskers, most extreme observations within 1.5 times the interquartile range from the box limits.

Figure 2 shows learning curves and overall average hit rates. Hit rates were higher under atomoxetine (gain: *M* = 0.66, SD = 0.27; loss: *M* = 0.67, SD = 0.18) compared to placebo (gain: *M* = 0.63, SD = 0.23; loss: *M* = 0.57, SD = 0.17). This difference was significant for the loss condition (*β* = −0.25, *χ*^2^ = 4.44, *p* = 0.046), but not for the gain condition (*β* = −0.03, *χ*^2^ = 0.02, *p* = 0.907). Effects of session order were not significant (gain: *β* = −0.16, *χ*^2^ = 0.47, *p* = 0.531; loss: *β* = −0.24, *χ*^2^ = 4.17, *p* = 0.071). Mean response times did not differ significantly between the atomoxetine (gain: *M* = 1.00 s, SD = 0.23 s; loss: *M* = 1.11 s, SD = 0.28 s) and placebo (gain: *M* = 1.03 s, SD = 0.23 s; loss: *M* = 1.11 s, SD = 0.27 s) sessions [gain: *F*(1, 17) = 1.33, *p* = 0.265; loss: *F*(1, 17.02) = 0.03, *p* = 0.862)].

### Reinforcement learning modelling

We applied a Q-learning model separately for the gain and loss conditions, using hierarchical Bayesian estimation to fit the model to the observed task data (Methods; Fig. 1b). The two parameters modelled were learning rate ($\alpha$) and inverse temperature ($\beta$). Higher values of $\alpha$ correspond to greater emphasis on recent outcomes and stronger discounting of earlier outcomes for determining the *Q*-value. Higher values of $\beta$ correspond to more deterministic or exploitative actions that are consistent with the expected values, whereas lower $\beta$ values correspond to more random or exploratory actions.

#### Individual parameter group-level estimate comparisons

**Fig. 3:**
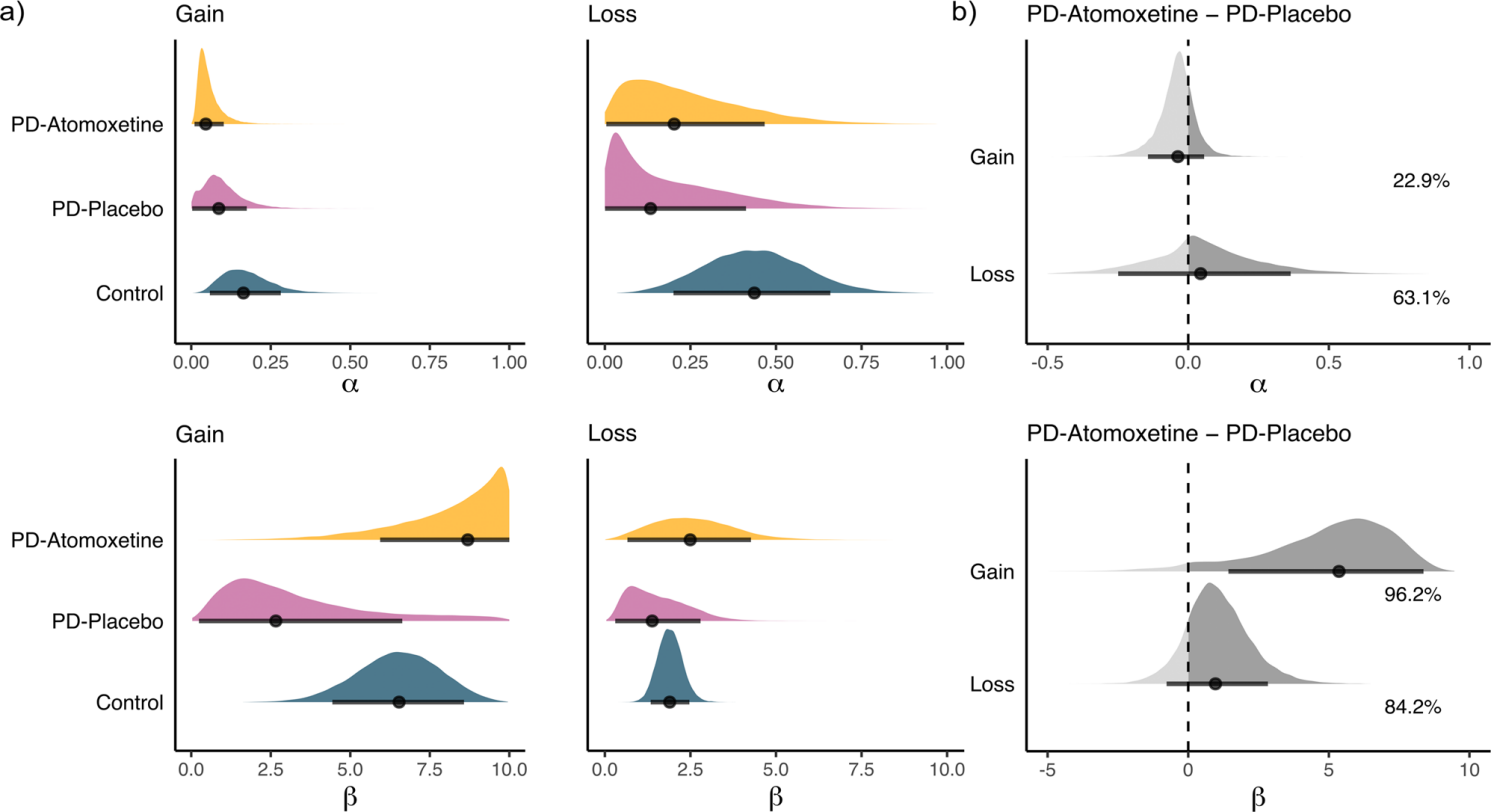
Group-level reinforcement learning parameters. **a** *Group-level reinforcement learning parameters*: Estimated posterior distributions of group-level means of learning rate (*α*) and inverse temperature (*β*) parameters. **b** *Group-level contrasts*: Posterior distributions of group-level means of drug contrasts, i.e., comparison of the distributions between atomoxetine *minus* placebo. The dark grey sections and percentage annotations denote the proportion of the distribution that was positive. Black dots represent the median and the black line segment represents the 89% highest density interval (HDI).

The estimated posterior distributions of group-level means of the parameters $\alpha$ and $\beta$ are shown in Fig. 3a. To examine drug effects on the parameters, we subtracted the posterior samples of the two drug-state conditions—i.e., atomoxetine *minus* placebo (Fig. 3b). The probability of direction (*p*_dir_) indicates the proportion of the distribution that was strictly positive or negative, whichever was greater. The region of practical equivalence (ROPE) corresponds to a negligible effect size. We report the percentage of the whole posterior distribution contained within the ROPE. Smaller proportions inside the ROPE equate to greater evidence against the null hypothesis and vice versa, with values < 2.5% in favour of rejecting the null hypothesis and values > 97.5% in favour of accepting the null hypothesis. We report medians as the point estimate of the posterior and the 89% highest density interval (HDI) as the credible interval.

For the learning rate α, the estimated group-level means generally did not suggest a strong drug effect (Fig. 3a, top row; Fig. 3b, top panel). In the gain condition, the mean α estimates were relatively low, both under atomoxetine (median = 0.05; 89% HDI [0.01, 0.10]) and placebo (median = 0.09; 89% HDI [0.003, 0.17]). The estimated drug effect, ∆*α*_gain_, was not clearly different from zero (median = −0.04; 89% HDI [-0.14, 0.06]), with a *p*_dir_ of 77.13% and 5.73% in the ROPE. Similarly, in the loss condition, the mean α estimates were comparable under atomoxetine (median = 0.20; 89% HDI: [0.004, 0.47]) and placebo (median = 0.13; 89% HDI: [0.0006, 0.41]); the estimated drug effect, ∆*α*_loss_, was broadly distributed around zero (median = 0.04; 89% HDI [−0.25, 0.37]) with a *p*_dir_ of 63.12% and 10.84% in the ROPE.

Larger differences were apparent for the inverse temperature *β*, with generally higher *β* values under atomoxetine than placebo (Fig. 3a, bottom row; Fig. 3b, bottom panel). In the gain condition, the mean *β* estimates were considerably higher under atomoxetine (median = 8.69; 89% HDI: [5.92, 10.00]) compared to placebo (median = 2.66; 89% HDI: [0.24, 6.63]). The estimated drug effect, ∆*β*_gain_, was clearly positive (median = 5.36; 89% HDI [1.43, 8.37]), with a *p*_dir_ of 96.24% and 1.35% in the ROPE. Qualitatively speaking, the same pattern was evident in the loss condition, to a lesser extent, with higher mean *β* estimates under atomoxetine (median = 2.49; 89% HDI: [0.66, 4.27]) compared to placebo (median = 1.38; 89% HDI: [0.30, 2.79]). The estimated drug effect, ∆*β*_loss_, was positive (median = 0.97; 89% HDI [−0.77, 2.83]) with a *p*_dir_ of 84.15% and 9.26% in the ROPE.

**Fig. 4:**
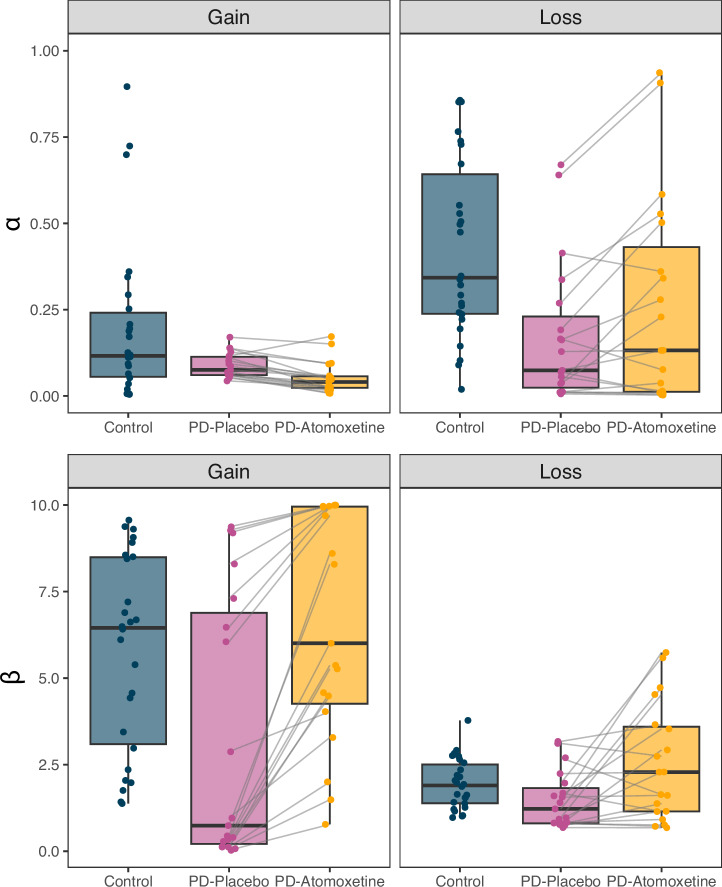
Participant-level reinforcement learning parameters. Point estimates (posterior median) of participant-level learning rate (*α*; top row) and inverse temperature (*β*; bottom row) parameters, plotted separately for each task condition (columns) and group/drug condition (colour). Individual participants are represented by dots; grey lines represent within-participant change between placebo and atomoxetine drug conditions (*n* = 19 PD-atomoxetine/PD-placebo; *n* = 26 controls). Box plot elements: centre line, median; box limits, first and third quartiles; whiskers, most extreme observations within 1.5 times the interquartile range from the box limits.

We present point estimates (posterior medians) of the participant-level *α* and *β* estimates (Fig. 4) to provide a visual intuition of participant-level changes on atomoxetine versus placebo. However, we refrain from further analysis of these parameter estimates, given concerns about biased test statistics resulting from post-hoc analysis of hierarchically estimated model parameters (see the “Methods” section for details).

#### Joint parameter space group-level estimates

Analysing the model parameters *α* and *β* separately overlooks the dependency between these parameters and their joint contribution to behaviour. To address this, we examined the relationship between *α* and *β* within their joint parameter space. Using a simulation procedure outlined in ref. ^37^, we first identified the “optimal” set of *α* and *β* values for both the gain and loss conditions of our task (full details in Supplementary Material). Here, “optimal” refers to the combination of *α* and *β* parameter values that maximised the hit rate. Next, we plotted the posterior distributions of the group-level means of *α* and *β* in their joint parameter space, resulting in two-dimensional heatmaps of parameter values for each experimental condition. We then examined these estimated joint parameter spaces with respect to the optimal joint parameter values (based on the simulation). Specifically, for each MCMC sample, we calculated the Euclidean distance between the estimated and optimal parameter set, yielding posterior distributions of the Euclidean distance for each experimental condition.

**Fig. 5:**
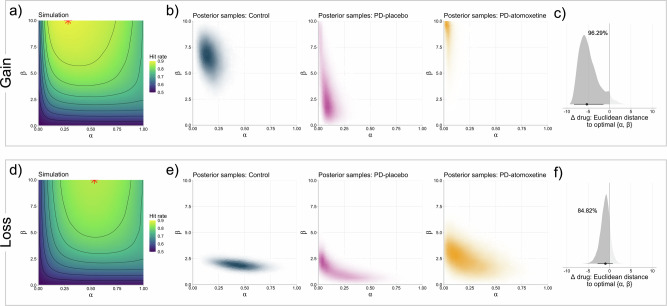
Joint parameter space. **a, d** *Simulated hit rates for all possible combinations of α and β*: Red asterisk denotes the “optimal” parameter combination that yielded the highest simulated hit rate; the contours and colour scale refer to the underlying density distribution of the hit rate estimates, with the contours dividing the range of that data into equally spaced bins; **b, e** *Estimated posterior distributions of group-level means of α and β in the joint parameter space*: The two-dimensional density of a given point in the joint parameter space is represented by the colour hue, with darker hues corresponding to greater posterior density. For clarity, points with the lowest observed two-dimensional density (typically corresponding to combinations of α and β that only occurred in a single MCMC sample) are plotted in white, rendering them invisible. **c, f** *Drug effects on the Euclidean distance between estimated and “optimal” joint parameter values*: Posterior distributions of the drug effect (i.e., difference between atomoxetine and placebo) on the Euclidean distance between the estimated group-level means of *α* and *β* and the optimal *α* and *β*. The dark grey sections and percentage annotations denote the proportion of the distribution that was negative (i.e., reduced distance to the optimal values). Black dots represent the median and the black line segment represents the 89% highest density interval (HDI).

The results of these analyses are illustrated in Fig. 5. For both the gain and loss conditions, the simulation procedure (Fig. 5a, d) revealed that the optimal *β* values were at the maximal values (here, 10), consistent with the high inverse temperatures that are expected in a stable, fixed-reward learning environment^37^. Optimal α values were lower in the gain condition compared to the loss condition, in keeping with previous results using this task in healthy people^38^. In the gain condition, it was evident that the estimated joint parameter space was closer to the optimal values on atomoxetine than placebo (Fig. 5b). The drug effect on the Euclidean distance was clearly negative (Fig. 5c; median = −5.32; 89% HDI [−8.32, −1.43]; *p*_dir_ = 96.29%), suggesting that atomoxetine reduced the distance to the optimal parameter values relative to placebo. The same pattern was observed in the loss condition, but to a lesser extent (Fig. 5e), with a slightly reduced Euclidean distance between the estimated joint parameter space and the optimal values on atomoxetine compared to placebo (Fig. 5f; median = −0.97; 89% HDI [−2.81, 0.77]; *p*_dir_ = 84.28%).

### Pupillometry

We subsequently examined pupillometry data recorded during the reinforcement learning task to obtain putative proxy measures of tonic and phasic activity of the locus coeruleus noradrenaline system. Analysis focused on pupil diameter in the baseline period as a measure of responsivity to the drug (i.e., tonic noradrenaline levels), and the temporal derivative in the first 1000 ms of the outcome phase of the task as a measure of the phasic pupil response to feedback. Baseline pupil refers to a 500 ms pre-trial epoch during the fixation period, from which the median pupil diameter value was extracted. We computed the first-order temporal derivative of the pupil signal in the outcome phase, that is, the signed difference between each timepoint and the previous timepoint.

#### Baseline pupil diameter

**Fig. 6:**
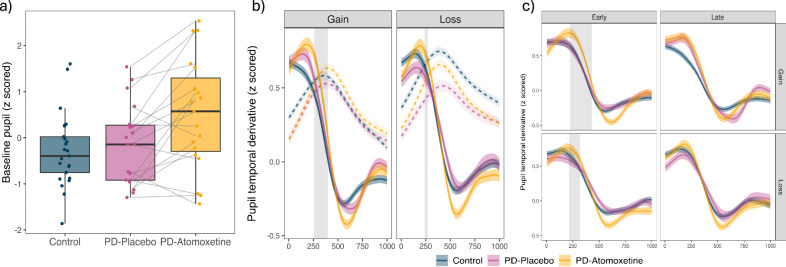
Baseline pupil and pupil temporal derivative during outcome phase of the task. **a** *Baseline pupil diameter*: Median pupil diameter during a 500 ms epoch in the fixation phase, averaged across all trials and task conditions, and plotted separately for each group / drug condition. Individual participants are represented by dots; grey lines represent within-participant change between placebo and atomoxetine drug-state conditions (*n* = 19 PD-atomoxetine/PD-placebo; *n* = 26 controls). Box plot elements: centre line, median; box limits, first and third quartiles; whiskers, most extreme observations within 1.5 times the interquartile range from the box limits. **b** *Temporal derivative of pupil signal in the initial 1000* *ms window of the outcome phase*: Solid lines show group averages of the temporal derivative, and ribbons represent standard error of the mean across trials. Grey bars highlight the group difference in initial phasic response detected by the cluster analysis. Dashed line shows the pupil diameter during this period. The x-axis represents the time (ms) since the start of the outcome phase. **c** *Pupil temporal derivative for early and late task trials*: Temporal derivative of pupil signal in the initial 1000 ms window, for the first (early) and second (late) half of the task trials. All visual elements are analogous to those in panel **b**.

Figure 6a shows the baseline pupil, averaged across trials and across the gain/loss conditions. Under atomoxetine, participants had significantly larger pupil diameters compared to placebo [*F*(1, 17) = 10.73, *p* = 0.004], with no effect of visit order [*F*(1, 17) = 0.22, *p* = 0.647].

#### Phasic pupil response to outcome feedback

Cluster-based permutation testing was conducted on the pupil temporal derivative for the first 1000 ms of the outcome feedback phase (Fig. 6b). In the gain condition, there was an increased phasic pupil response under atomoxetine (atomoxetine > placebo: cluster = 259–397 ms, cluster mass statistic (CMS) = 535.79, *p* < 0.001). This was accompanied by a later cluster where values were higher in the placebo group (placebo > atomoxetine: cluster = 547–619 ms, CMS = 175.79, *p* < 0.001). In the loss condition, there was also an increased phasic pupil response under atomoxetine (atomoxetine > placebo: cluster = 239–271 ms, CMS = 68.89, *p* < 0.001). This was followed by a later cluster where values were higher in the placebo group (placebo > atomoxetine: cluster = 409–623 ms, CMS = 1044.89, *p* < 0.001).

#### Early trials vs. later trials

Cluster-level inference was conducted on the temporal derivative signal separately for the first half and second half of the task trials (Fig. 6c). In the gain condition the initial phasic response under atomoxetine was evident in the first half of the task (atomoxetine > placebo: cluster = 223–431 ms, CMS = 968.68, *p* < 0.001). This was accompanied by a later cluster (placebo > atomoxetine: cluster = 531–699 ms, CMS = 991.38, *p* < 0.001) where values were higher in the placebo group. The initial phasic difference was not apparent in the second half of the task, with significant clusters only in the later period (atomoxetine > placebo: cluster = 667–753 ms, CMS = 251.63, *p* < 0.001; placebo > atomoxetine: cluster = 897–949 ms, CMS = 120.73, *p* < 0.001).

In the loss condition, the initial phasic response under atomoxetine was evident in the first half of the task (atomoxetine > placebo: cluster = 213–317 ms, CMS = 387.20, *p* < 0.001), followed by a later difference (placebo > atomoxetine: cluster = 423–647 ms, CMS = 1037.65, *p* < 0.001). The initial phasic difference was absent in the second half of the task, with only a later difference (placebo > atomoxetine: cluster = 415–553 ms, CMS = 428.92, *p* < 0.001).

### Associations between atomoxetine effects on baseline pupil and reinforcement learning

**Fig. 7:**
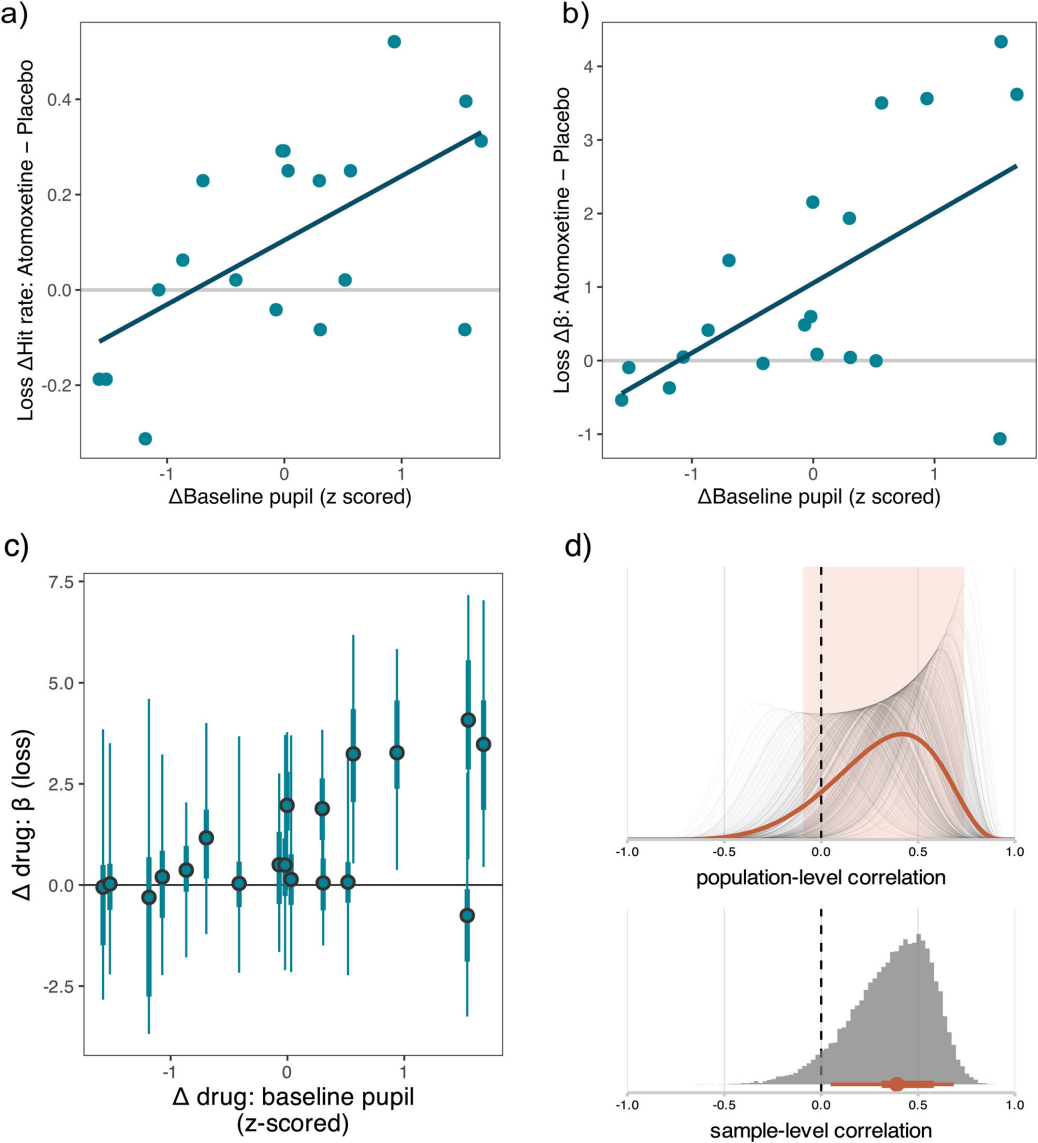
Associations between atomoxetine effects on baseline pupil and reinforcement learning. Relationship between atomoxetine-induced change in baseline pupil diameter and loss hit rate (**a**) and loss *β* estimates (**b**). Each dot represents a given Parkinson’s disease participant’s atomoxetine effect (i.e., atomoxetine *minus* placebo) on the loss hit rate (**a**) and loss *β* parameter estimate (**b**) (*y*-axis). This captures how their performance changed under atomoxetine, with values above the grey line indicating an increase in values under the drug. The *x*-axis shows the atomoxetine-induced change in baseline pupil (i.e., atomoxetine *minus* placebo), with positive values indicating an increase in baseline pupil under the drug. **c** Atomoxetine-induced change in baseline pupil diameter and loss *β* estimates, where the thin line elements represent the 89% HDI, thick line elements represent the 50% HDI, and dots represent posterior medians of the atomoxetine effect on the *β* parameter estimate in the loss condition. **d** Plausible values analysis of the correlation between baseline pupil and loss *β* drug effects. Bottom panel: For each MCMC sample, the Pearson correlation between the atomoxetine effects on loss *β* estimates and baseline pupil diameter was computed, yielding a distribution of plausible correlation coefficients for the current sample of participants (grey histogram). The orange interval plot represents the 89% HDI (thin line), 50% HDI (thick line) and median (point) (*n* = 19). Top panel: For each of the plausible correlation coefficients, a posterior distribution was computed based on the analytic solution given in Ly et al.^39^ (thin grey lines). The mean of these posterior distributions (orange line) provides a summary for inference on the correlation coefficient in the population. The light-orange background represents the 89% HDI of the mean posterior distribution. To avoid overplotting, individual posterior density traces have been plotted for a subset of 500 MCMC samples.

Lastly, we examined whether individual differences in the pupil-derived responsivity to atomoxetine predicted its effects on reinforcement learning, both in terms of task performance and model parameters (Fig. 7). Specifically, we used the change in median baseline pupil diameter (i.e., Δ baseline pupil: atomoxetine−placebo) as an index of drug responsivity related to tonic noradrenaline levels. We then tested whether this individual difference measure was associated with atomoxetine-induced changes in (i) observed hit rate, and (ii) estimated learning rate (*α*) and inverse temperature (*β*).

To test for an association with hit rate, we fit a linear mixed model separately for the gain and loss conditions, with hit rate as the dependent variable and drug condition, baseline pupil diameter change score and their interaction as fixed effects, and session order as a covariate of no interest (see the “Methods” section for details). There was a significant interaction between drug state and the baseline pupil diameter change score on hit rate [*F*(1, 16) = 9.99, *p* = 0.006, *p*_fdr_ = 0.012], specifically in the loss condition. This reflected a positive correlation between the atomoxetine-induced changes in baseline pupil diameter and hit rate in the loss condition (*r* = 0.60, *t*(17) = 3.09, *p* = 0.007, BF = 8.36; Fig. 7a). In other words, participants with a greater baseline pupillary response to atomoxetine tended to have greater atomoxetine-induced increases in hit rate in the loss condition. There was no drug × Δ baseline pupil interaction effect for hit rate in the gain condition (*p* = 0.599).

To assess associations between atomoxetine-induced changes in baseline pupil diameter and the estimated parameters *α* and *β*, we used the “plausible values” correlation method proposed by Ly et al. ^39^ (see the “Methods” section for details). In brief, this method addresses concerns about post-hoc analysis of hierarchically estimated parameters by incorporating uncertainty at two levels: (i) the estimation uncertainty of participant-level parameter values, and (ii) the sampling uncertainty associated with generalising from the observed sample of participants to the population. For a given correlation analysis, this method provides a distribution of plausible correlation coefficients within the current sample (“sample-level correlation”), as well as a posterior distribution of the latent correlation in the population (“population-level correlation”).

We found credible evidence for a positive correlation between atomoxetine-induced changes in baseline pupil diameter and *β* estimates in the loss condition (sample-level correlation coefficient: median = 0.39, 89% HDI [0.05, 0.68]; *p*_dir_ = 94.07%; Fig. 7b, c). This suggests that participants with a greater baseline pupillary response to atomoxetine tended to have greater increases in *β* in the loss condition. The population-level estimate of the correlation coefficient supported this effect, though the credible interval included zero (population-level correlation coefficient: median = 0.34, 89% HDI [−0.09, 0.74]; *p*_dir_ = 86.62%; Fig. 7d).

A similar, though weaker, association with *β* was observed in the gain condition, with correlation estimates at both the sample-level (median = 0.24, 89% HDI [−0.05, 0.52]; *p*_dir_ = 88.90%) and population-level (median = 0.21, 89% HDI [−0.21, 0.61]; *p*_dir_ = 76.70%) yielding insufficient evidence for a credible effect. No meaningful associations were found between baseline pupillary changes and α estimates, in either the gain (sample-level: median = 0.07, 89% HDI [−0.32, 0.42]; *p*_dir_ = 62.59%) or loss (sample-level: median = 0.02, 89% HDI [−0.38, 0.39]; *p*_dir_ = 52.78%) conditions.

## Discussion

Noradrenaline can be a powerful modulator of learning. In the context of Parkinson’s disease, we confirm that reinforcement learning behaviour is improved after a single 40 mg dose of the noradrenaline reuptake inhibitor atomoxetine. The nature of the improvement included higher hit rates under atomoxetine and parameter estimates that more closely resembled healthy controls and optimal simulations. Elevated phasic pupil responses under atomoxetine and links with baseline pupil add further weight to a noradrenergic, rather than a confounding dopaminergic, mechanism driving these improvements in learning behaviour.

For the model parameters, the most prominent changes under atomoxetine relative to placebo were in inverse temperature (*β*). Higher *β* values were seen under atomoxetine in both the gain and loss conditions, corresponding to more deterministic, exploitative choices that are consistent with expected outcomes. This was in the direction of *β* estimates in optimal simulations and in the control group (bearing in mind that comparisons with unmedicated controls should be made cautiously). In a relatively stable environment, such as the task under investigation, following initial exploration to identify the optimal course of action, an adaptive agent should converge on an exploitative strategy. This is supported by simulations showing that high *β* values are optimal in a probabilistic fixed reward setting (Fig. 5a, d)^37^. In line with our result, reinforcement learning in healthy cohorts under volatile conditions^19^ and when information-seeking is manipulated^40^, also shows an effect of 40 mg atomoxetine on parameters quantifying explore-exploit (akin to inverse temperature). Learning rates (*α*) were slightly higher under atomoxetine compared to placebo for the loss condition; similarly, these were in the direction of α estimates for healthy controls and the optimal simulations. In the joint parameter space, estimates under atomoxetine were closer to optimal parameter values compared to placebo (Fig. 5c, f).

Taken together, our modelling results suggest a change in learning behaviour under atomoxetine—consistent with learning behaviour that was more similar to controls and to simulations of “optimal” learning strategies. Atomoxetine was also associated with increased baseline pupil diameter and increased pupil responses in the outcome phase. Baseline pupil diameter is a proxy for overall brain state effects on arousal over the longer term^41^, which has been linked to tonic noradrenergic activity^36,42^. A robust increase in baseline pupil diameter has been observed following a single dose of 40 mg atomoxetine in young healthy people^19^, suggesting that our finding is not specific to Parkinson’s disease. We found that a greater baseline pupil change under atomoxetine was associated with increased hit rates and *β* estimates in the loss condition (Fig. 7). And while we did not see this relationship across both conditions, it suggests that responsivity to the drug in terms of increased arousal/increased noradrenaline levels (as indexed by baseline pupil) was beneficial for task performance.

To examine task-evoked pupil response to outcome feedback, we used the pupil derivative (i.e., rate of change), where positive values reflect pupil dilation and negative values constriction^43^. Fast evoked pupil dilations have been associated with phasic locus coeruleus activity^42,44^. Across all groups, we show a biphasic pupil response after receiving feedback, with early dilation followed by later constriction, which was amplified under atomoxetine compared to placebo. A biphasic pupil response to feedback replicates work in healthy young participants using a probabilistic reinforcement learning task^45^. Our results do not disambiguate a functional interpretation of the increased phasic pupil response under atomoxetine. However, transient locus coeruleus-noradrenergic activation is observed following reinforcement delivery during learned behaviour, and has been associated with negative and positive prediction error^23^ and shown to causally influence performance in subsequent trials^6^, with these signals transmitted to cortical projection sites^6,23^. This positions feedback-driven locus coeruleus activation as broadcasting a widespread signal to sculpt learning from reinforcers and bias subsequent behaviour. In our task, phasic pupil response to outcome feedback may be consistent with new learning, driven by both the salience of the feedback (i.e., it is highly informative for the task at hand) and the unexpected outcomes due to the probabilistic nature of the task. The locus coeruleus responds to novel stimuli, but this response habituates^46^. The pattern we observed, where the difference in phasic pupil response between atomoxetine and placebo was apparent in the early trials and absent in later trials, may be consistent with habituation. When learning has occurred, feedback is less informative and outcomes less surprising—and therefore less likely to engage the locus coeruleus-noradrenergic system in the later phases of the task.

Our findings challenge a simplistic application of existing models of locus coeruleus-noradrenergic activation—largely derived from animal work—to human disease data. Consistent with a non-linear “inverted-U” relationship between noradrenergic status and behaviour, performance should be optimal at moderate tonic levels and pronounced phasic activity, with suboptimal performance when tonic levels are high or low (and phasic activity is reduced)^24^. However, our pupil data are consistent with both increased baseline (tonic) levels and increased phasic response on atomoxetine, in the context of better task performance. An important caveat in extrapolating the tonic-phasic activation framework to human data is that baseline/tonic measures are typically taken while participants are engaged in performing a task (e.g., the pre-stimulus period); this likely creates a state closer to a “phasic mode”^41^. In the context of disease states that affect locus coeruleus-noradrenergic function, we would add the additional caveat: the inverted-U relationships between chemical neuromodulator levels and performance are offset by Parkinson’s disease, to a different degree across different brain regions^47^. This means that optimal neuromodulator levels for a given task are likely to deviate from those in healthy participants to a degree that depends on the functional anatomical correlates of the task (as is the case for dopaminergic functions^47^). Indeed, for our Parkinson’s patients in the atomoxetine drug state, while their learning performance converged towards that of controls, visual comparison of their baseline and outcome-related pupil responses showed clear differences from controls. Together, these points suggest caution in inferring a high tonic locus coeruleus activation mode from baseline pupil data and highlight that in disorders with multisystem changes (including noradrenergic and autonomic), the relationships between peripheral markers and cognitive function mapped out in healthy people may not operate in the same way.

Reinforcement learning deficits in medicated Parkinson’s disease initially led to a “dopamine overdose” theory, whereby elevated dopamine levels in ventral fronto-striatal circuitry impaired certain cognitive functions^48,49^. This was subsequently related to the valence of reinforcement: unmedicated patients were commonly impaired at learning from positive feedback due to a lack of dopamine signalling reward, and medicated patients were impaired at learning from negative feedback as elevated dopamine obscured dips that signal reward absence^50^. In contrast to this, under atomoxetine, we observed beneficial effects on learning performance across both gain and loss conditions. This highlights a compelling clinical implication of our work: the benefits of atomoxetine may help alleviate some of the learning deficits that persist with, or are exacerbated by, dopamine medication. Mechanisms by which dopamine and noradrenergic medication might interact in Parkinson’s disease remain to be elucidated. Noradrenaline is proposed to preserve behavioural accuracy in the face of high-dopaminergic activational states, effectively curbing a performance deficit that might occur when behaviour is invigorated^51,52^, with evidence for such opponency occurring at the nucleus accumbens^53^. This line of evidence raises the possibility that augmenting noradrenaline in Parkinson’s disease might also lead indirectly to cognitive benefits by curbing the deleterious impact of dopamine over-activation.

Failures to replicate dopamine-related asymmetric learning effects on reward vs. punishment in Parkinson’s disease (e.g. refs. ^54,55^) have prompted increasing recognition that phenotypic variation may underscore considerable differences in dopamine-based learning deficits^56–58^. Our findings highlight the need to consider noradrenergic contributions to individual variability in learning deficits, in line with mounting evidence that this currently untreated system has a pervasive impact on cognitive-psychiatric features in Parkinson’s disease and related conditions^2,59,60^. Another future avenue would be to explore gender differences in performance, noting that our control and patient samples both had an unbalanced gender distribution—with a higher proportion of males consistent with a higher prevalence of Parkinson’s in males. Future work in larger, gender-matched samples could examine possible gender differences in atomoxetine-induced changes in learning.

We emphasise a noradrenergic interpretation of our findings for two key reasons: (1) atomoxetine is not likely to strongly modulate the striatal dopaminergic circuitry implicated in Parkinson’s disease reinforcement deficits, given the paucity of striatal noradrenergic projections and negligible action of the noradrenaline transporter there^34^; (2) the modulation of both baseline and phasic pupil responses under the drug, which are closely linked with noradrenergic activity. While we cannot rule out possible effects of elevated prefrontal dopamine levels that can occur under atomoxetine, activation at the nucleus accumbens shell (which is innervated by the medullary A2 noradrenergic cell population of the nucleus tractus solitarius^61^) or indirect activations given the extensive interactions between the noradrenaline and dopamine systems^62^, our results contrast starkly with previous dopaminergic investigations of reinforcement learning in Parkinson’s disease: namely, the drug improved behaviour in both gain and loss conditions. Our findings support a distinct role for noradrenergic modulation of learning in Parkinson’s disease, consistent with its well-established role in normative neuroscience models of learning.

In summary, our findings extend noradrenergic frameworks of reinforcement learning into a Parkinson’s disease model. We show improvements in learning strategy following noradrenergic modulation, with prominent effects on the parameter governing explore-exploit. These improvements were in the context of baseline and phasic pupil changes under the drug, substantiating a noradrenergic mechanism driving the behavioural change. In turn, the findings demonstrate that noradrenergic therapy is a viable approach to treat cognitive symptoms in Parkinson’s disease and related disorders.

## Methods

The general study design and participant characteristics overlap with previous publications^63–66^. The study was approved by the Cambridge Research Ethics Committee (REC 10/H0308/34) and participants provided written informed consent. The study is registered on the ISRCTN registry with study ID ISRCTN46299660. The study was retrospectively registered because it was exempt from Clinical Trials status by the UK Medicines and Healthcare Products Regulatory Authority (MHRA). All ethical regulations relevant to human research participants were followed.

### Participants

Nineteen people with idiopathic Parkinson’s disease were recruited via the University of Cambridge Parkinson’s disease research clinic and the Parkinson’s UK volunteer network. All participants met the United Kingdom Parkinson’s Disease Society Brain Bank criteria, were aged between 50–80 years, with Hoehn and Yahr stages 1.5–3, and had no contraindications to 7 T MRI or atomoxetine. No participant met clinical criteria for dementia or had an impulse control disorder. Twenty-six age-, sex- and education-matched healthy control participants were recruited. Control participants were screened for a history of psychiatric or neurological disorders, and were not taking psychoactive medications.

### Study procedure

Participants with Parkinson’s disease were tested over three sessions. In the first session, they completed MRI scanning and clinical assessment of cognition and motor function (Table 1; extended clinical information reported in Supplementary Material, Section 2 “Additional clinical information”). Sessions two and three were a double-blind, randomised, placebo-controlled crossover design, with 40 mg oral atomoxetine or placebo. These visits were ≥6 days apart (mean 7.4; standard deviation 1.7; range 6–14). Blood samples were taken two hours after administration of the drug/placebo to coincide with the predicted peak plasma concentration of atomoxetine after a single oral dose^67^. Mean plasma concentration^68^ was 261.32 ng/mL after atomoxetine (standard deviation 117.33 ng/mL, range 90.92–595.11 ng/mL) and 0 ng/mL after placebo. After the blood sample, patients commenced an experimental task battery, including the reinforcement learning task. Blood pressure and pulse rate were taken across the sessions, and visual analogue scales were administered to monitor mood/arousal levels (see Supplementary Table 4; Supplementary Fig. 6). All sessions were completed at a similar time of day, with participants on their regular anti-Parkinsonian medications. Control participants provided reference data, and they were tested in a single session without drug manipulation.

### Reinforcement learning task

The task was based on established reinforcement learning paradigms involving two pairs of visual stimuli probabilistically associated with monetary gains or losses^38,69^. One pair formed the gain condition, where stimuli were associated with either a 75% probability of winning 50 pence or a 25% probability of winning nothing. The other pair formed the loss condition, where stimuli were associated with a 75% probability of losing 50 pence or a 25% probability of losing nothing. In the gain condition, participants should learn to select the symbol associated with the 75% win probability (i.e., choose the correct symbol). In the loss condition, participants should learn to avoid the symbol associated with the 75% loss probability (i.e., avoid choosing the incorrect symbol).

The task was programmed in Python (version 2.7). Participants responded using lightly sprung keyboard buttons marked ‘L’ and ‘R’ to indicate the left or right stimulus. The stimuli were two-dimensional shapes (Fig. 1a). Experimental trials used standard geometric shapes that were familiar and easy to distinguish; the practice session used less familiar shapes (e.g., hourglass, circular sector) to avoid confusion with the test stimuli. To increase discriminability between the gain and loss conditions, in a counterbalanced order across participants, in one condition, the shapes were formed of a single line and in the other condition, they were formed of a double line. All stimuli, including the fixation cross and outcomes, were matched for mean luminance and contrast using the SHINE toolbox implemented in MATLAB R2018b^70^.

Each trial involved a fixation cross presented for 3.5 s, followed by the shape stimuli presented until the participant responded or for a maximum of 2.5 s (if participants failed to respond, the message “Too late” was displayed). Outcome feedback was then presented for 2.5 s. The long duration of the fixation cross allowed time for the pupil to return to baseline levels following the outcome. See Fig. 1a for an example of the trial structure.

Participants were seated in front of a laptop with a chin rest to prevent head movement. Following task instructions, a 3-point calibration routine was performed using the Eyelink software, after which participants performed a practice session of 24 trials. The experiment consisted of 96 trials in total, with 48 trials from the gain condition and 48 trials from the loss condition. The order of gain and loss trials was randomised within blocks of 12 trials. Participants were given two brief rest breaks during the task. Alternate versions of the task were created using different shapes, and the order of the versions was counterbalanced across participants’ first and second visits.

Behavioural performance on the task (i.e., accuracy and response time) was analysed using linear mixed models, with response accuracy modelled using a binomial distribution due to binary response data. We obtained *p*-values using a parametric bootstrap (binomial data) or using the Kenward–Roger method.

### Reinforcement learning modelling

#### Q-learning model

We applied a *Q*-learning model separately for the gain and loss conditions, as both Parkinson’s disease^50^ and catecholaminergic manipulations^29,38,69^ can differentially affect reinforcement learning parameters under positive vs. negative feedback. The *Q*-learning model assumes participants form an expected value for each stimulus and corresponding action (i.e., the *Q*-value), and update this value based on the feedback received in each trial. The model consists of two components: (i) a *learning* rule, which describes how feedback is used to update the expected values, and (ii) a *choice* rule, which describes how the expected values are translated into actions.

We used the Rescorla–Wagner “delta” rule as the learning rule. For each trial $t$, an action $a$ is performed towards a stimulus $s$, which yields an outcome $r$. Based on this outcome, the *Q*-value of the respective action for the given stimulus is updated for the upcoming trial $t+1$ as follows:

1 $${Q}_{t+1}\left({s}_{t},{a}_{t}\right)={Q}_{t}\left({s}_{t},{a}_{t}\right)+\alpha \left[{{r}_{t}-Q}_{t}\left({s}_{t},{a}_{t}\right)\right]$$

where $\alpha$ ($\alpha \in \left[{{\mathrm{0,1}}}\right]$) is the learning rate, which determines to what extent the *Q*-value is adjusted by the prediction error—that is, the difference between the observed outcome and the expected value. The influence of the learning rate $\alpha$ on expected values is illustrated in Fig. 1b. Higher values of $\alpha$ correspond to greater emphasis on recent outcomes and stronger discounting of earlier outcomes for determining the *Q*-value. In the extreme case of $\alpha =1$, the *Q* values are fully determined by the observed outcomes; by contrast, with $\alpha =0$, no learning occurs and the *Q*-values are fixed to initial values.

The choice rule is described by the softmax function, which translates *Q*-values into action probabilities. Specifically, given a stimulus $s$, the probability of performing action $a$, and not alternative action ${a}^{{\prime} }$, is defined as follows:

2 $$P({a}_{t}|{s}_{t})=\frac{\exp \left[\beta \times {Q}_{t}\left({s}_{t},{a}_{t}\right)\right]}{\exp \left[\beta \times {Q}_{t}\left({s}_{t},{a}_{t}\right)\right]+\exp \left[\beta \times {Q}_{t}\left({s}_{t},{a}_{t}^{{\prime} }\right)\right]}$$

where $\beta$ ($\beta \in [{{\mathrm{0,10}}}]$) is the inverse temperature, which determines the level of noise or stochasticity in action selection. If $Q(s,a)$ gradually increases while $Q(s,{a}^{{\prime} })$ is held constant, the action probabilities will gradually tend towards $P\left(a,|,s\right)=1$ and $P\left({a}^{{\prime} },|,s\right)=0$. Put simply, performing an action becomes increasingly likely if its expected value becomes increasingly high relative to other options. The influence of the inverse temperature $\beta$ on action probabilities is illustrated in Fig. 1b. Higher values of $\beta$ correspond to more deterministic or exploitative actions that are consistent with the expected values, whereas lower $\beta$ values correspond to more random or exploratory actions. In the extreme case of $\beta =0$, action selection is completely random and independent of expected values.

#### Model implementation

We used hierarchical Bayesian estimation to fit the model to the observed task data, separately for the control group and Parkinson’s disease group. Hierarchical fitting methods assume that participant-level parameters are sampled from corresponding group-level distributions, which helps regularise participant-level parameter estimates and enables reliable group-level inference^71^. We employed non-centred parameterisation, which is recommended in smaller datasets^72^. This means that for each free parameter, we estimated the group-level mean $\mu$ and standard deviation $\sigma$, as well as participant-wise deviations from the mean $\nu$. Parameter bounds were enforced by transforming parameter values from the real line $\left(-\infty ,\infty \right)$ to the probability scale $\left[{{\mathrm{0,1}}}\right]$ using the cumulative distribution function of the standard normal distribution, denoted $\Phi \left(\cdot \right)$. Taken together, for a given participant and task condition (gain or loss), the parameters $\alpha$ and $\beta$ were estimated as follows:

3 $${\alpha }^{{\prime} }={\mu }_{\alpha }+{\sigma }_{\alpha }\times {\nu }_{\alpha }$$

4 $${\beta }^{{\prime} }={\mu }_{\beta }+{\sigma }_{\beta }\times {\nu }_{\beta }$$

5 $$\alpha =\Phi \left({\alpha }^{{\prime} }\right)$$

6 $$\beta =\Phi \left({\beta }^{{\prime} }\right)\times 10$$

where ${\alpha }^{{\prime} }$ and ${\beta }^{{\prime} }$ denote the untransformed parameter values.

For the Parkinson’s disease group, we additionally accounted for drug effects on $\alpha$ and $\beta$ by estimating the change ($\Delta$) in these parameters on atomoxetine relative to placebo^31,73,74^. The parameters for the placebo condition were estimated as usual (Eqs. (3)–(6), and the parameters for the atomoxetine condition were estimated by shifting the placebo estimates with the drug “offset” values. Taken together, for the Parkinson’s disease group, the model parameters for a given participant and task condition (gain or loss) were estimated for each drug condition $i$ as follows:

7 $${\Delta }_{\alpha }={\mu }_{{\Delta }_{\alpha }}+{\sigma }_{{\Delta }_{\alpha }}\times {\nu }_{{\Delta }_{\alpha }}$$

8 $${\Delta }_{\beta }={\mu }_{{\Delta }_{\beta }}+{\sigma }_{{\Delta }_{\beta }}\times {\nu }_{{\Delta }_{\beta }}$$

9 $${\alpha }_{i}=\left\{\begin{array}{cc}\Phi \left({\alpha }^{{\prime} }\right),\hfill &{{{\rm{if}}}}i={{{\rm{placebo}}}}\hfill \\ \Phi \left({\alpha }^{{\prime} }+{\Delta }_{\alpha }\right),&{{{\rm{if}}}}i={{{\rm{atomoxetine}}}}\end{array}\right.$$

10 $${\beta }_{i}=\left\{\begin{array}{cc}\Phi \left({\beta }^{{\prime} }\right)\times 10,\hfill &{{{\rm{if}}}}i={{{\rm{placebo}}}}\hfill \\ \Phi \left({\beta }^{{\prime} }+{\Delta }_{\beta }\right)\times 10,&{{{\rm{if}}}}i={{{\rm{atomoxetine}}}}\end{array}\right.$$

We assigned the standard normal distribution (i.e., normal distribution with mean of 0 and standard deviation of 1) as the prior distribution for all group-level means $\mu$ and all participant-wise deviations from the means $\nu$. We assigned a half-normal distribution with a mean of 0 and standard deviation of 1 as the prior distribution for all group-level standard deviations $\sigma$.

The model was implemented in Stan and was run using the rstan interface package^75^ in R version 3.6.1. We used Stan’s “No-U-Turn Sampler” Markov chain Monte Carlo (MCMC) algorithm to sample from the joint posterior distribution of the model parameters. The sampling was performed with 4 chains of 10,000 iterations each, and the first 2000 iterations of each chain were discarded as the warmup. Model convergence was assessed with the potential scale reduction statistic $\hat{R}$ (<1.1 for all parameters), and with visual inspection of the MCMC trace plots (Supplementary Figs. 1, 2). To assess goodness of fit, we performed graphical posterior predictive checks (Supplementary Fig. 3).

#### Model comparison

We fit two additional models to verify that the standard two-parameter model adequately explained the data. One model contained only the learning rate and a parameter known as the lapse rate ($\epsilon$), which captures the proportion of random choices made irrespective of their expected value^76^. Another model contained all three free parameters ($\alpha ,\beta ,\epsilon$). The standard two-parameter model clearly outperformed the first model, and the three-parameter model offered no meaningful improvement in explaining the data, supporting our parsimonious choice of the two-parameter model. Model comparison was performed using approximate leave-one-out cross-validation. These comparisons are described in the Supplementary Material (Supplementary Table 1; Supplementary Fig. 4).

#### Model parameter analysis

For group-level analysis, we derived posterior distributions for group contrasts by subtracting the set of MCMC samples of the two groups under consideration. We report the probability of direction (*p*_dir_) as an index of *existence* of an effect and the region of practical equivalence (ROPE) as an index of *significance* of the effect^77^, implemented using bayestestR package^78^. The *p*_dir_ indicates the proportion of the distribution that is strictly positive or negative. The ROPE was set at: 0 ± 0.1 × SD^79^, where SD refers to the point estimate (posterior median) of the group-level standard deviation of the parameter under consideration. This range of ±0.1 in the standardised parameter space is a convention based on defining the negligible effect size at half of Cohen’s definition of a “small” effect (i.e., 0.2)^80^. Thus, the ROPE delineates a negligible effect size, i.e., the region practically equivalent to the null value. We report the percentage of the whole posterior distribution contained within the ROPE. Smaller proportions inside the ROPE equate to greater evidence against the null hypothesis and vice versa, with values < 2.5% in favour of rejecting the null hypothesis and values > 97.5% in favour of accepting the null hypothesis^78^. We report medians as the point estimate of the posterior and the 89% highest density interval (HDI)^81^ as the credible interval. The HDI is the range of values that contains the most probable proportion (here, 89%) of the posterior of a given parameter, which directly characterises the (un)certainty of an estimate^80^. The 89% interval was chosen as this is deemed to be more numerically stable than the commonly used 95% interval^81,82^.

For participant-level parameters, we provide a graphical overview of point estimates (i.e., posterior medians) across the different groups as well as drug and task conditions (Fig. 4), but refrain from further inferential statistics based on these estimates. Since participant-level parameters were estimated hierarchically, each participant’s data informed the others to regularise parameter values, resulting in estimates that are “shrunk” towards the group-level mean^83^. While this improves the average accuracy of the participant-level estimates^84^, it complicates post-hoc statistical testing. Specifically, a post-hoc analysis typically requires a single summary value (i.e., point estimate) of a parameter for each participant and each cell of the experimental design. This ignores the within-participant posterior uncertainty of parameter values, as well as the between-participant dependency imposed by the hierarchical modelling structure. Consequently, the empirical variance of the point estimates underestimates the true between-participant variance, which can result in biased test statistics^85^. Thus, we emphasise the group-level analyses of parameter means for inference on the effects of atomoxetine.

### Pupillometry

#### Preprocessing

Pupil data were sampled at 500 Hz using an Eyelink Portable Duo, with participants’ heads stabilised on a chin rest. Preprocessing was performed using custom Python scripts. Blinks under 500 ms were linearly interpolated with interpolation spanning 100 ms before and after the blink^86^; where gaps were longer than 500 ms the trial was discarded^87^. Second-order low-pass Butterworth filtering was applied. For each trial, a pre-trial baseline period was created using the final 500 ms of the fixation period. To prevent extreme baseline values from influencing the derived pupil values, trials that contained baseline values ± 2 standard deviations from an individual’s mean within a session were removed^88^. Baseline correction was then applied by subtracting the median pupil value from the final 500 ms of the fixation period from each pupil diameter value in a trial^88^. The first-order temporal derivative of the pupil signal for the 1000 ms of the outcome phase was computed. The rate of change in pupil diameter (i.e., its temporal derivative) rather than absolute change in pupil diameter, can be a better predictor of phasic noradrenergic activity, cortical state and task performance^44,89,90^.

#### Pupil analysis

For the baseline analysis, median values from each baseline period were averaged across trials and across the gain/loss conditions to create a summary value for each person. Gain and loss trials were collapsed here, as the baseline period was agnostic to trial condition (see Supplementary Material, Supplementary Fig. 10, for comparisons showing that the baseline periods following gain vs. loss trials did not differ, further supporting our approach to combine them). Baseline pupil values were *z*-scored prior to analysis and compared using linear mixed models. Drug effects were analysed using linear mixed models (see “Statistics and reproducibility” section below for details).

To compare the pupil temporal derivative across groups for the first 1000 ms of the outcome phase, we used cluster-based permutation testing. This analysis was conducted separately for the gain and loss conditions, using pupil temporal derivative values *z*-scored by subject, within each session (i.e., placebo, atomoxetine), and averaged across trials for each participant. Cluster-based permutation tests for linear mixed models were implemented using the clusterperm.lmer function from the R package permutes^91^. This method calculates *t*-statistics at each time point. Consecutive *t*-statistics above a threshold are summed to create a cluster mass statistic (CMS), which is then compared against a permuted null distribution to generate *p*-values. The permutes package infers the permuted null distribution using Likelihood Ratio Tests. We modelled participants as random effects and used 2000 permutations, specified in R syntax as follows: clusterperm.lmer(Temporal derivative ~ Drug + (Drug|Subject)), where Drug is placebo vs. atomoxetine. To explore whether group differences in the phasic pupil response were more prominent during the early learning phase of the task, we conducted the same analysis on individuals’ averaged pupil derivatives from the first half of the trials and the second half of the trials separately.

### Participant-level model parameters and pupillometry

To examine associations between atomoxetine effects on pupil responses and estimated model parameters (*α* and *β*), we used a “plausible values” correlation analysis method introduced by Ly et al.^39,92^ and implemented in the R package plausiblecor^93^. This method enables reliable inference about the correlation between a latent model parameter—estimated using hierarchical Bayesian methods—and an observed covariate. It was specifically developed to address concerns about biased test statistics arising from post-hoc analysis of hierarchically estimated parameters. First, to account for the estimation uncertainty of participant-level parameters, the Pearson correlation coefficient is computed for each MCMC sample. This yields a distribution of plausible correlation coefficients, as opposed to a single correlation coefficient based on point estimates of the model parameter. Second, for each plausible correlation coefficient, a posterior distribution is obtained that reflects the uncertainty of generalising from the observed sample to the population. Specifically, an approximation of the unnormalised posterior density function is obtained by computing the posterior density for an equally-spaced grid of correlation values, based on the analytic solution provided by Ly et al.^92^ These separate posterior distributions are then averaged to obtain a mean posterior distribution of the correlation coefficient. According to Ly et al.^39^, the distribution of plausible correlation coefficients is suitable for inference on the correlation in the observed sample of participants; the mean posterior distribution of the correlation coefficient is suitable for inference on the latent correlation in the population.

We performed the plausible values correlation analysis separately for atomoxetine effects on *α* and *β* in the gain and loss task conditions. Each of these model parameters was examined with the atomoxetine effect on baseline pupil diameter as the covariate of interest. We focus on this pupil measure as a putative index of responsivity to atomoxetine.

### Statistics and reproducibility

Across all analyses, we compared the same Parkinson’s disease patients when they were on placebo vs. when they were on atomoxetine (we refer to this as differences in drug state). Data from healthy controls is visually presented alongside to provide normative data to benchmark performance in the patient groups. For all main outcome measures, we have included analyses of healthy controls vs. Parkinson’s on placebo to quantify the disease effect, noting that the difference lies not only in the presence of disease but also placebo effects and task order effects in people with Parkinson’s disease. These are presented in Supplementary Material Section 6 “Healthy control vs. Parkinson’s disease placebo”.

Where linear mixed models were used, these were implemented in R version 4.2.1 using the afex package. A random intercept for participants was always included, with session order included as a fixed effect of no interest; where possible random slopes for the fixed effects of interest were included [e.g., Hit rate ~ Drug*Condition + Session + (Condition|Subject) + (Drug|Subject), where Drug is placebo vs. atomoxetine; Condition is gain vs. loss; Session is visit order 1st vs. 2nd]. If this model produced a singular fit, random effects and covariance structure were reduced to the point where the model remained identifiable and produced qualitatively similar results to the full model)^94^ [e.g., Hit rate ~ Drug*Condition + Session +(0 + Condition|Subject)]. We obtained *p*-values for the regression coefficients using the Kenward–Roger method. The emmeans package was used for post hoc comparisons.

### Reporting summary

Further information on research design is available in the Nature Portfolio Reporting Summary linked to this article.

## Supplementary information

Supplementary Material

Reporting Summary

## Data Availability

Data to reproduce manuscript figures and analysis are openly available to download here https://osf.io/7ez5r/.

## Code availability

Stan model code and the code to reproduce manuscript figures and analysis are openly available here https://osf.io/7ez5r/.
