# Supplementary material for: Pharmacological and pupillary evidence for the noradrenergic contribution to reinforcement learning in Parkinson’s disease
Source: Commun Biol. 2025 Aug 14;8:1223. doi: 10.1038/s42003-025-08627-2 (PMC12354755; doi:10.1038/s42003-025-08627-2)
Supplement: Supplementary file 1 — Supplementary Material [file 42003_2025_8627_MOESM1_ESM.pdf]

Supplementary material

Pharmacological and pupillary evidence for the noradrenergic contribution to reinforcement learning in Parkinson's disease

1. Reinforcement learning model validation
i) MCMC trace plots Page 2
ii) Posterior predictive checks Page 4
iii) Model comparisons Page 5

2. Additional clinical information
i) ACE-R and UPDRS subscales Page 6
ii) Mood and behaviour questionnaires Page 6
iii) Within session physiological effects Page 8
iv) Within session subjective effects Page 10

4. Healthy control vs. Parkinson's disease placebo
i) Reinforcement learning task performance Page 12
ii) Reinforcement learning task modelling Page 12
iii) Pupillometry Page 13

5. Joint parameter simulation Page 14

6. Baseline pupil gain vs. loss Page 15

7. References Page 16

MCMC trace plots

Below are the Markov chain Monte Carlo (MCMC) trace plots for the α, β reinforcement model that was the focus of the study. These are time series plots for the group level means of the parameters alpha and beta, showing the evolution of a parameter value across iterations of the Markov chain. The diagnostic plots below suggest the model has converged as the chains have mixed and resemble hairy caterpillars. Plotted using the *bayesplot* package[1] implemented in R. The greyed area denotes the burn-in period of 2000 samples.

Gain condition

Supplementary Figure 1

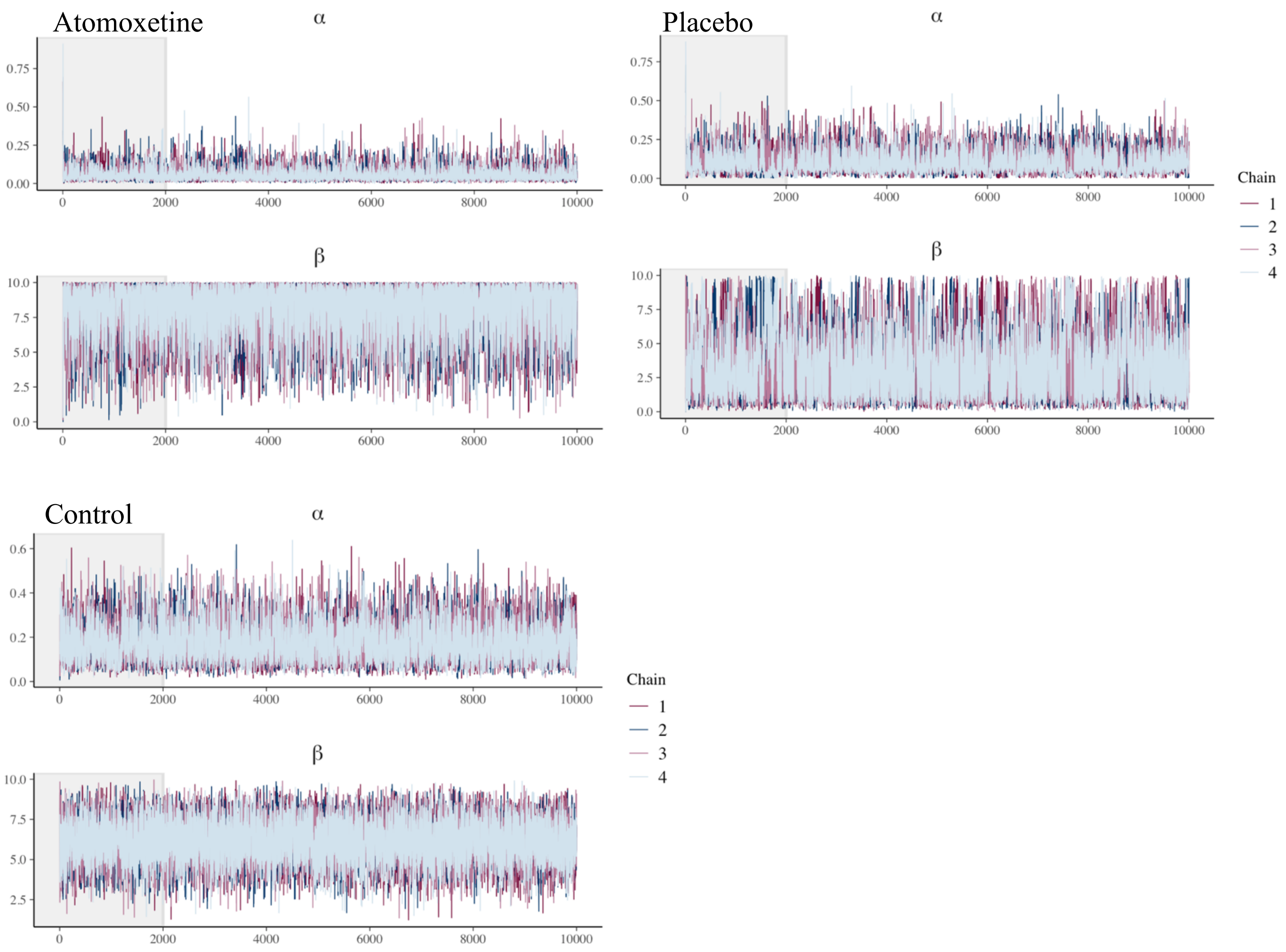

Loss condition

Supplementary Figure 2

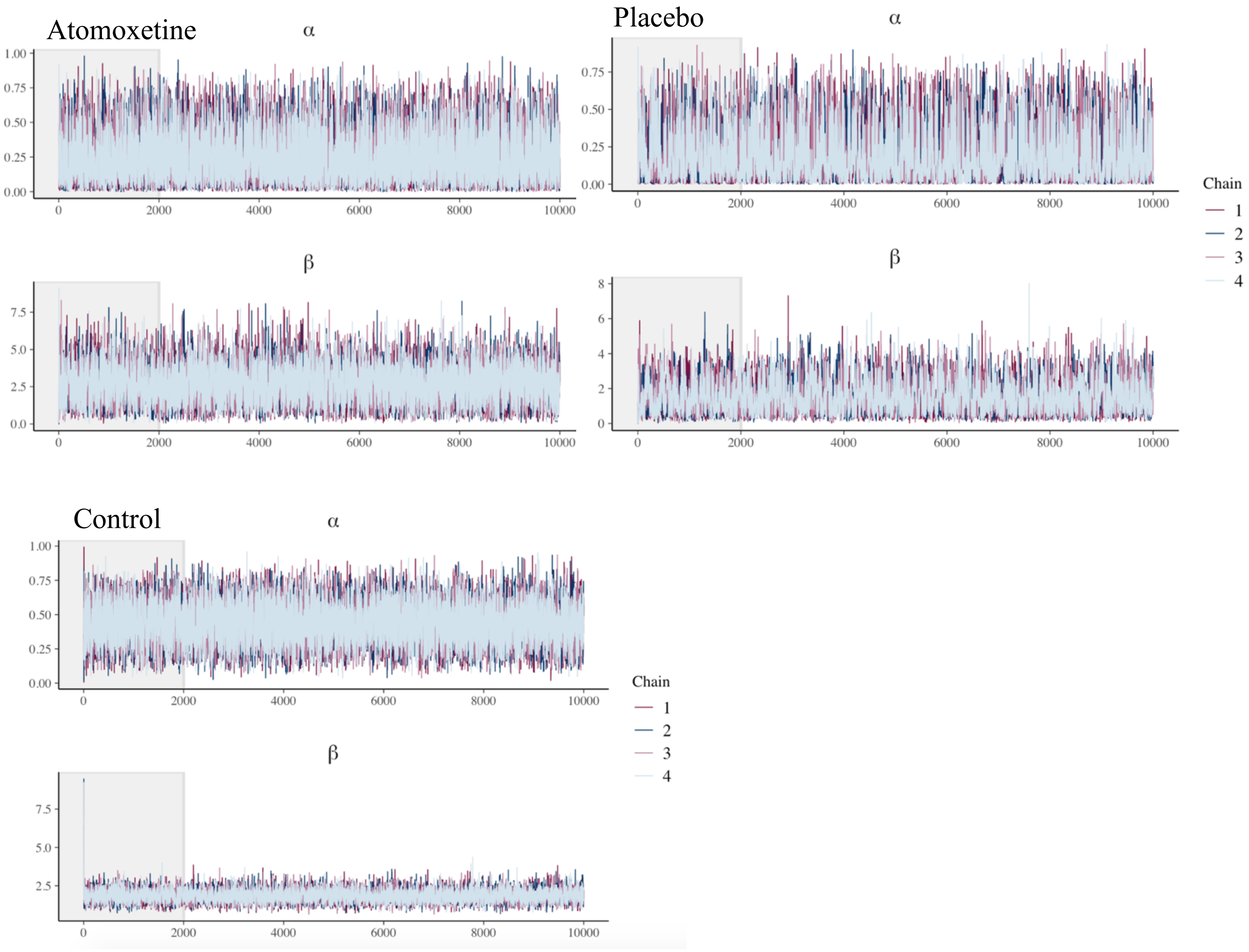

Posterior predictive checks

We used Stan's `generated quantities` block in our model code to simulate a full dataset of outcome values (i.e., choices) from the model's posterior predictive distribution, for each iteration of model fitting. The posterior predictive distribution is the distribution of the outcome variable implied by a model after using the observed data to update beliefs about the unobservable model parameters. Intuitively, if a model is a good fit, then simulated data drawn from the model's posterior predictive distribution should closely resemble the observed data. We used the *bayesplot* R package[1] for graphical comparisons between the observed data (y) and the simulated data (y^{rep}; i.e., simulated replications of y).

Supplementary Figure 3

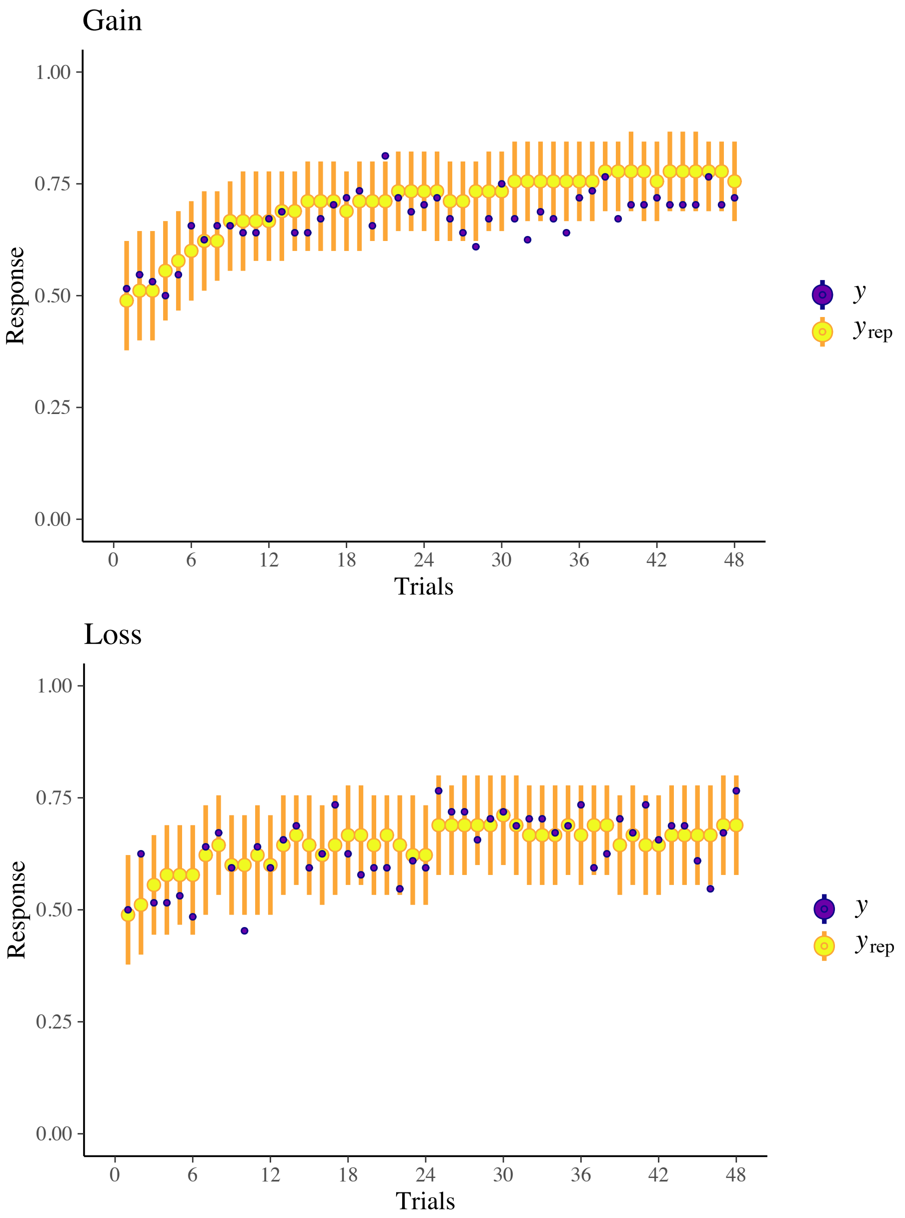

Supplementary Figure 3 – Overall mean response values for observed data (y) against mean response values of simulated data (y^{rep}); thick orange lines show 90% probability interval.

Model comparisons

We used Stan's `generated quantities` block in our model code to save the trial-wise log likelihood, for each iteration of model fitting. Using the *loo* R package[2] we compared expected log predictive density (ELPD) estimates of the three models. The difference in ELPD is computed relative to the model with the highest ELPD. Differences between models can be considered meaningful when ELPD differences are > 4 and where the difference in ELPD is larger than twice the estimated standard error[3]. Model 1 is the two parameter learning rate and inverse temperature model (α, β). Model two contained only the learning rate and lapse rate parameters (α, ε). Model three contained all three free parameters ($\alpha, \beta, \varepsilon$). Both Models 1 and 3 outperformed Model 2; ELPD differences were negligible between Models 3 and 1, with a difference much less than 2 × SE. Together supporting Model 1 as the best performing and most parsimonious choice.

Supplementary Table 1

Model comparisons

condition	model	elpd_diff	se_diff	elpd_loo	se_elpd_loo	p_loo	se_p_loo	looic	se_looic
**gain**	**model3**	**0.0000000**	**0.000000**	**-1421.131**	**26.39441**	**59.95613**	**4.6748426**	**2842.263**	**52.78881**
gain	model1	-0.3348153	2.579203	-1421.466	26.92308	60.80021	5.0833428	2842.933	53.84616
gain	model2	-375.7970195	19.254805	-1796.929	11.57368	21.83521	0.6080112	3593.857	23.14736
**loss**	**model3**	**0.0000000**	**0.000000**	**-1819.369**	**22.20943**	**75.86235**	**3.2093926**	**3638.739**	**44.41886**
loss	model1	-0.9924185	2.281000	-1820.362	22.60245	73.89128	3.5709580	3640.723	45.20489
loss	model2	-93.2026087	13.157179	-1912.572	13.01034	20.65148	0.5083706	3825.144	26.02068

Supplementary Table 1 – Loo results are sorted in the order with best performing model in the top. This model is then used as the common comparison point.

Supplementary Figure 4

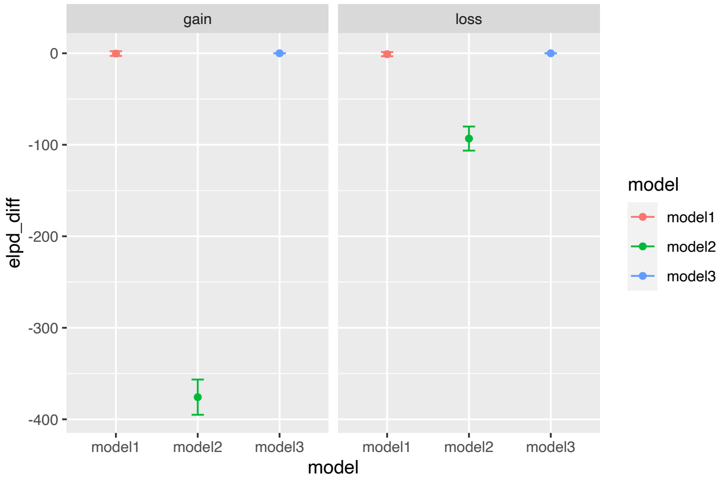

Supplementary Figure 4 – Plot of *loo* comparisons, with error bars showing standard error of the ELPD difference.

ACE-R and MDS-UPDRS subscales

The Table below shows additional details for the Movement Disorders Society Unified Parkinson's Disease Rating Scale (MDS-UPDRS)[4] and the revised Addenbrooke's cognitive examination (ACE-R)[5]. Patients had a significantly lower ACE-R total score (p = .015), and lower memory subscale (p = .013).

Supplementary Table 2

		PD	Controls	*p*
ACE-R	Total Score	94.89 (3.71)	97.58 (3.16)	**.015**
	Attention & Orientation	17.84 (0.37)	17.96 (0.20)	.216
	Memory	23.68 (1.97)	25.04 (1.18)	**.013**
	Fluency	12.00 (2.08)	12.81 (1.60)	.167
	Language	25.84 (0.50)	25.88 (0.43)	.768
	Visuospatial	15.63 (0.50)	15.81 (0.63)	.302
MDS-UPDRS	I: Nonmotor experiences	9.00 (4.18)		
	II: Motor experiences	12.63 (4.26)		
	III: Motor Examination	28.42 (11.60)		
	IV: Motor Complications	0.47 (0.96)		
	Total Score	50.58 (17.20)		

Mood and behaviour questionnaires

Individuals with Parkinson's disease completed self-rated questionnaires to assess mood and behavioural symptoms. These included assessment of anxiety and depression (Hospital Anxiety and Depression scale; HADS)[6], impulsivity (Barratt Impulsiveness Scale; BIS-11[7]; Conners' Adult ADHD Rating Scale; CAARS)[8], apathy (Apathy Scale[9]; Motivation and Energy Inventory; MEI)[10] and REM sleep behaviour disorder (REM sleep behaviour disorder screening questionnaire; RBDSQ[11]. Controls also completed all of these self-rated questionnaires, apart from the RBDSQ. Informant-rated questionnaires were collected from a relative or friend of the patients. These included informant versions of the CAARS and AS, and a general mood and behaviour symptom inventory (Cambridge Behavioural Inventory Revised; CBI-R[12].

Supplementary Table 3

**Measure**		**PD**	**Controls**	***p***
Apathy Scale	Total Score (self-rated)	12.68 (5.77)	10.58 (5.09)	.212
	Total Score (informant-rated)	13.13 (5.59)		
BIS	Total Score	56.45 (10.34)	56.15 (9.67)	.924
	Attention	14.16 (4.3)	14.23 (3.72)	.953
	Motor	20.08 (2.65)	20.85 (3.38)	.398
	Non-planning	22.21 (5.4)	21.08 (4.44)	.459
HADS	Anxiety	4.53 (3.2)	4.31 (3.53)	.83
	Depression	3.95 (2.68)	2.88 (2.76)	.202
MEI	Total Score	98.05 (21.3)	108.96 (16.71)	.073
	Mental	44.11 (8.97)	47.35 (8.09)	.22
	Physical	23.95 (6.95)	29.35 (5.91)	**.01**
	Social	30 (7.34)	32.27 (5.31)	.261
CAARS (self-rated)	Inattention / Memory Problems	5.42 (3.58)	4.19 (3.06)	.235
	Hyperactivity / Restlessness	3.11 (2.71)	2.77 (2.05)	.652
	Impulsivity / Emotional Lability	1.84 (1.5)	2.77 (2.05)	.087
	Problems with Self-Concept	2.26 (2.33)	3.88 (4.12)	.102
	ADHD Index	6.79 (4.26)	8.38 (4.51)	.233
CAARS (observer-rated)	Inattention / Memory Problems	4.24 (2.51)		
	Hyperactivity / Restlessness	1.58 (1.92)		
	Impulsivity / Emotional Lability	1.21 (1.23)		
	Problems with Self-Concept	2.32 (2.11)		
	ADHD Index	4.47 (3.42)		
RBDSQ		4.58 (3.45)		
CBI	Total Score	15.13 (13.6)		
	Abnormal Behaviour	0.84 (1.12)		
	Beliefs	0.37 (1.21)		
	Eating Habits	0.95 (1.58)		
	Everyday Skills	1.16 (2.41)		
	Memory and Orientation	4.66 (3.9)		
	Mood	1.26 (2.1)		

Motivation	2.26 (3.35)
Stereotypic and Motor Behaviours	0.79 (1.4)
Self Care	0.42 (0.84)
Sleep	2.42 (2.17)

Note: Data are presented as mean (SD). Group comparisons were performed with independent samples t-tests; *p*, two-tailed *p*-values, uncorrected for multiple comparisons.

Supplementary Figure 5

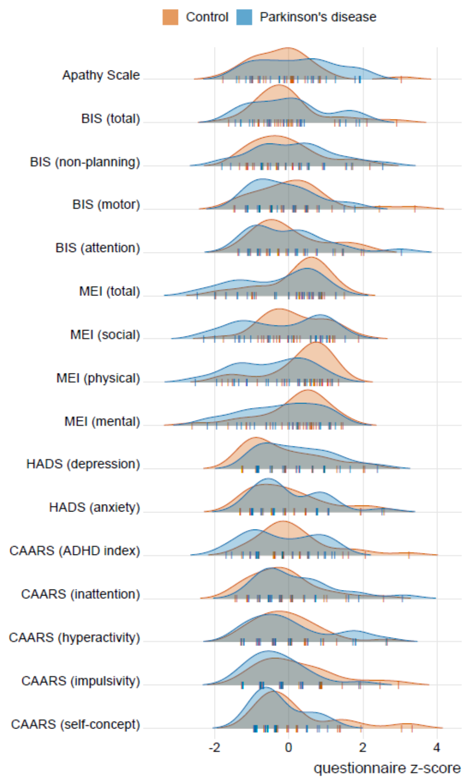

Supplementary Figure 5 – Density plots of questionnaire outcomes for the patient (blue) and control (orange) groups. Each questionnaire outcome was z-scored to facilitate visual comparisons of different questionnaires (note that group comparisons for a given questionnaire outcome are unaffected by this transformation). Tick marks reflect individual data points.

Within session physiological effects

Within the drug and placebo sessions, there was some evidence of increased pulse rates and raised blood pressure on atomoxetine, although this was not considered clinically relevant and was not consistently observed across all supine and upright measures.

As described below, there was evidence of increased pulse rates under atomoxetine, when assessed in the upright (but not the supine) position. Systolic and diastolic blood pressure was also increased under atomoxetine for supine (but not upright) measures. There was evidence for a time effect on supine blood pressure measures, where they were raised at completion of testing, relative to the measures on arrival and two-hours post tablet administration. Mean values and ranges for blood pressure and pulse rates are shown in Supplementary Table 4.

Pulse rates

Supine/Lying down pulse rates did not change significantly under atomoxetine vs. placebo, as evidenced by a lack of main effect ($F_{(1, 89)} = 5.57$, $p = .020$), and did not vary across the three time points (i.e., arrival, two-hours post tablet, on completion of testing; $F_{(2, 89)} = 3.00$, $p = .055$. Upright pulse rates were increased under atomoxetine, showing a significant main effect ($F_{(1, 88.03)} = 20.99$, $p < .001$), and a significant interaction between drug status and time point ($F_{(2, 88.03)} = 6.43$, $p = .002$) driven by higher pulse rates under atomoxetine at two hours post administration ($t_{(88)} = 10.93$, $p < .001$) and on completion of testing ($t_{(88)} = 10.39$, $p < .001$).

Blood pressure

Supine systolic and diastolic blood pressure was raised under atomoxetine, as evidenced by significant main effects (systolic: $F_{(1, 89)} = 9.86$, $p = .002$; diastolic: $F_{(1, 89)} = 16.21$, $p < .001$). Upright systolic and diastolic blood pressure did not show an overall change under atomoxetine, as evidenced by the lack of main effects (systolic: $F_{(1, 88)} = 2.41$, $p = .124$; diastolic: $F_{(1, 88)} = 0.00$, $p = .972$). However, there was a main effect of time point (systolic: $F_{(1, 88)} = 5.86$, $p = .004$; diastolic: $F_{(1, 88)} = 5.98$, $p = .004$), driven by increased blood pressure on completion of testing, compared to arrival and two hours post (systolic, arrival vs. completion: $t_{(88)} = 9.77$, $p = .011$; systolic, two hours post vs. completion: $t_{(88)} = 9.79$, $p = .010$; diastolic, arrival vs. completion: $t_{(88)} = 4.87$, $p = .012$; diastolic, two hours post vs. completion: $t_{(88)} = 5.60$, $p = .006$).

Supplementary Table 4

**Measure**			**Placebo**	**Atomoxetine**
Pulse rates	Lying	Arrival	70.00 (33.5 – 55.5; 8.85)	70.58 (56 – 91; 10.27)
		2-hours	69.95 (55 – 86; 10.37)	74.89 (58 – 95; 11.25)
		Completion	66.95 (50 – 85; 8.20)	70.21 (50 – 93; 11.06)
	Upright	Arrival	75.63 (49 – 100; 12.64)	74.95 (56 – 110; 14.30)
		2-hours	70.00 (54 – 86; 8.88)	80.95 (60 – 106; 13.91)
		Completion	68.33 (49 – 80; 7.88)	79.95 (57 – 116; 15.39)
Systolic blood pressure	Lying	Arrival	127.68 (84 – 151; 16.67)	133.26 (109 – 175; 17.24)
		2-hours	125.32 (95 – 156; 15.03)	135.11 (114 – 169; 15.35)
		Completion	131.21 (116 – 155; 12.54)	136.58 (83 – 186; 24.03)
	Upright	Arrival	124.37 (80 – 165; 21.78)	130.26 (90 – 166; 21.20)
		2-hours	123.21 (101 – 145; 12.47)	131.37 (93 – 167; 18.68)

		Completion	139.11 (118 – 176 14.60)	136.11 (97 – 183; 20.99)
Diastolic blood pressure	Lying	Arrival	70.89 (50 – 84; 8.61)	74.00 (55 – 85; 7.46)
		2-hours	68.37 (54 – 80; 6.68)	74.53 (58 – 94; 9.04)
		Completion	72.42 (59 – 87; 7.07)	77.21 (63 – 98; 9.93)
	Upright	Arrival	72.89 (43 – 82; 9.71)	73.32 (56 – 86; 8.09)
		2-hours	71.26 (51 – 91; 10.78)	73.47 (52 – 93; 9.82)
		Completion	79.44 (61 – 93; 8.15)	76.68 (61 – 92; 9.08)

Note: Data are presented as mean (range; SD).

Within session subjective effects

There was no change in subjective ratings of mood and arousal levels within the sessions. Although the visual analogue scale (VAS) is a continuous measure, participants often respond at either end of the scale, leading to bi- or even tri-modal distributions (Supplementary Figure 6). Such dynamics are not well captured by conventional analyses (e.g., linear regression) that assume multivariate normality. To address this issue, we analysed the VAS data using a Bayesian ordered beta regression model[13]. The strength of this model is that it simultaneously estimates the probability of responses at the scale's lower and upper bounds as well as continuously distributed responses in between the bounds.

Supplementary Figure 6

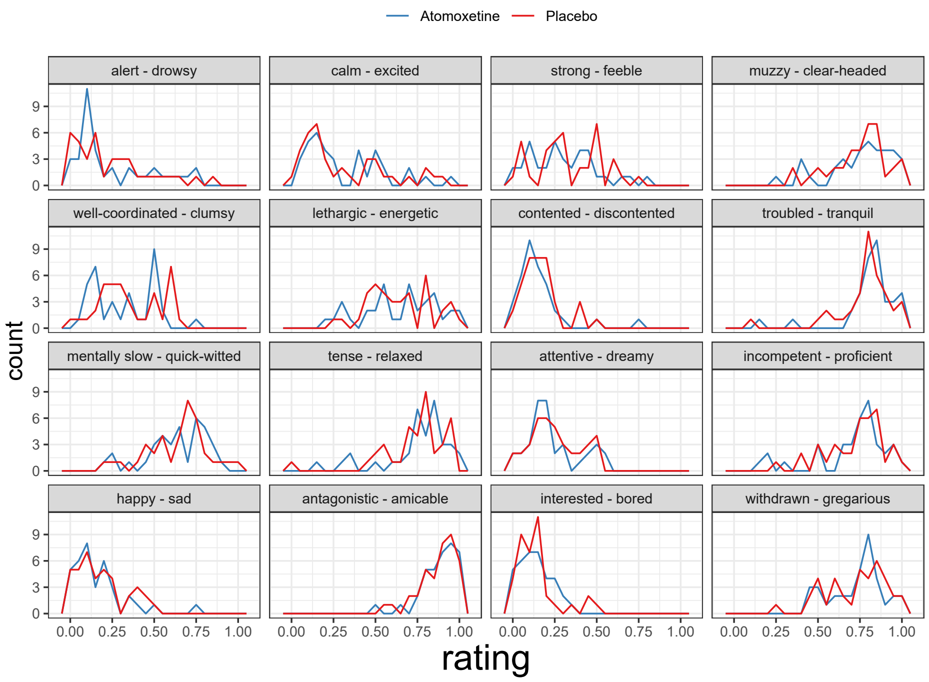

Supplementary Figure 6 – Frequency polygons of VAS ratings in the PD group. Each panel represents one VAS item, as described by the panel titles. The first term of each panel title corresponds to the left extreme of that VAS item (i.e. rating = 0), whereas the second term corresponds to the right extreme (i.e. rating = 1).

We modelled drug (atomoxetine vs. placebo), time point (2 hours post administration vs. baseline), the drug × time interaction, and session (first vs. second visit) as categorical predictors of the VAS response (i.e., fixed effects), and we allowed the intercept to vary by VAS item and by participant (i.e., random effects). Following Kubinec (2020), we assigned a weakly informative normal prior on the regression coefficients: $\beta \sim N(0, 5)$. For posterior inference, we set a region of practical equivalence (ROPE) at $\pm 0.1 \times SD_{VAS} = \pm 0.019$, corresponding to a negligible effect size[14,15].

There were no main effects of drug or time point on VAS response, as the posterior distributions of these coefficients were largely contained by the ROPE (Supplementary Figure 7; drug: β = -0.02, 95% HDI [-0.06, 0.03], proportion in ROPE = 46.20%; time point: β = 0.02, 95% HDI [-0.03, 0.06], proportion in ROPE = 49.69%). Although the posterior estimate of the drug × time point interaction effect was greater than the upper bound of the ROPE, we failed to reject the null as a relatively large proportion of the posterior distribution was contained by the ROPE (interaction: β = 0.03, 95% HDI [-0.01, 0.08], proportion in ROPE = 24.01%). Taken together, these results suggest that atomoxetine did not induce a significant change in subjective states, as measured by the VAS.

Supplementary Figure 7

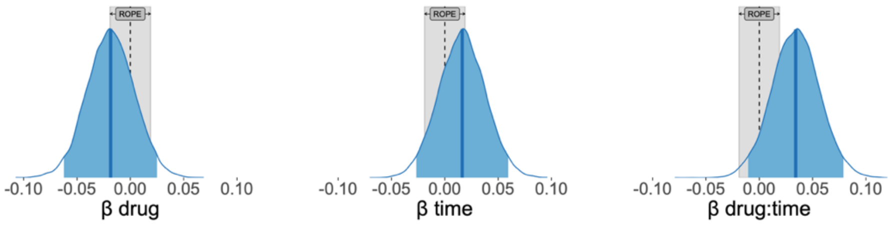

Supplementary Figure 7 – Posterior distributions of predictors of VAS responses. For each panel, the dark blue vertical line represents the median – that is, the posterior estimate of the regression coefficient; the blue shaded area represents the 95% highest density interval of the posterior distribution; and the blue density trace represents the full posterior distribution. The grey area represents a region of practical equivalence (ROPE), corresponding to a negligible effect size of ±0.1.

Healthy control vs. PD placebo – Reinforcement learning task performance

Control participants had higher hit rates than the placebo group (gain: M = 0.71, SD = 0.27; loss: M = 0.67, SD = 0.12), which were significantly different in the loss condition (gain: β = 0.20, $\chi^2 = 0.62$, $p = 0.464$; loss: β = 0.22, $\chi^2 = 4.95$, $p = 0.024$). Reaction times did not differ between the control (gain: M = 0.98 s, SD = 0.17 s; loss: M = 1.12 s, SD = 0.25 s) and placebo groups [gain: $F(1, 43) = 0.88$, $p = 0.354$; loss: $F(1, 43) = 0.02$, $p = 0.896$)].

Healthy control vs. PD placebo – Reinforcement learning task modelling

Individual parameters

For controls compared to placebo, controls had higher mean α and β estimates across both the gain and the loss conditions. Group level α estimates in controls (gain median = 0.16; 89% HDI: [0.06, 0.28]; loss median = 0.44; 89% HDI: [0.20, 0.66]) versus placebo indicated a stronger presence of effect in the loss condition [Δ Control α_{gain} - Placebo α_{gain}: median = 0.07; 89% HDI [-0.07, 0.23]; p_{dir} = 79.88%; Δ Control α_{loss} - Placebo α_{loss}: median = 0.28; 89% HDI [-0.10, 0.60]; p_{dir} = 87.21%]. Group level β estimates in controls (gain median = 6.53; 89% HDI: [4.44, 8.58]; loss median = 1.90; 89% HDI: [1.34, 2.47]) versus placebo indicated a stronger presence of effect in the gain condition [Δ Control β_{gain} - Placebo β_{gain}: median = 3.65; 89% HDI [-0.84, 7.27]; p_{dir} = 87.64%; Δ Control β_{loss} - Placebo β_{loss}: median = 0.49; 89% HDI [-1.06, 1.80]; p_{dir} = 67.83%].

Supplementary Figure 8

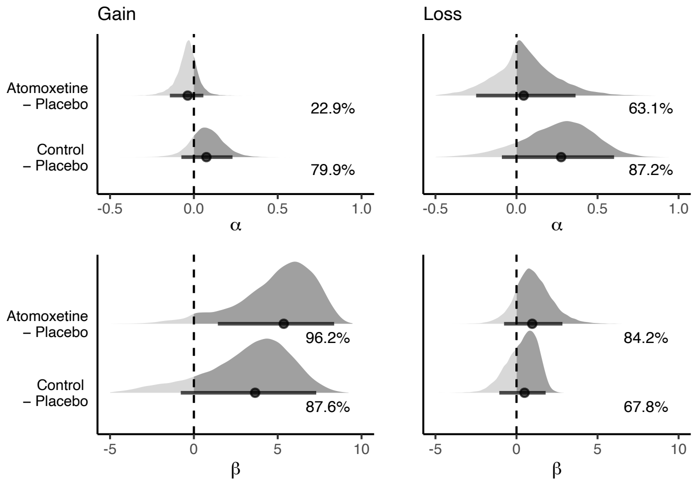

Supplementary Figure 8 – Group-level reinforcement learning parameters. **Posterior distributions of group-level means of drug contrasts and group contrasts**, i.e., comparison of the distributions between Atomoxetine *minus* Placebo and Controls *minus* Placebo. The dark grey sections and percentage annotations denote the proportion of the distribution that was positive. Black dots represent the median and the black line segment the 89% Highest Density Interval (HDI).

Joint parameter space

Compared to the PD placebo group (Figure 4b,e; Supplementary Figure 9, the controls generally had a smaller Euclidean distance between the estimated joint parameter space and the optimal parameter values, especially for the gain condition [Δ Control – Placebo for gain: median = -3.65; 89% HDI [-7.30, 0.81]; p_{dir} = 87.66%; Δ Control – Placebo for loss: median = -0.50; 89% HDI [-1.80, 1.04]; p_{dir} = 68.12%].

Supplementary Figure 9

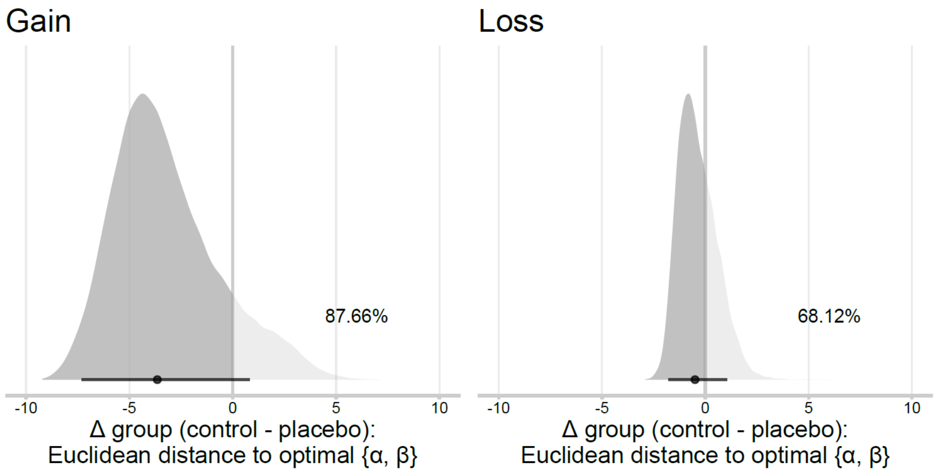

Supplementary Figure 9 – **Posterior distributions of group contrasts on Euclidean distance between estimated and "optimal" joint parameter values**, i.e., comparison of the distributions between Controls *minus* Placebo. The dark grey sections and percentage annotations denote the proportion of the distribution that was negative. Black dots represent the median and the black line segment the 89% Highest Density Interval (HDI).

Healthy control vs. PD placebo – Pupillometry

Baseline pupil

Baseline pupil diameter did not differ significantly between controls and participants on placebo [$F(1, 42) = 0.319$, $p = 0.575$].

Pupil temporal derivative cluster-level inference

No significantly different clusters were identified comparing controls and participants on placebo.

Joint parameter simulation

Using code adapted from[16], we simulated 1000 blocks of 48 trials for gain and loss conditions separately, using different combinations of α and β parameters. The parameter combinations were taken from a grid spanned by the learning rate (α; minimum value/maximum value/steps = 0/1/60) and inverse temperature β (0/10/60). Each of these virtual participants then completed a large number of trials (> 50,000) from a fixed probability learning task with a reward schedule of 75:25. For each parameter combination, hit rates associated with choosing the "correct" option were calculated. Following [16] to reduce random noise due to the finite number of samples, the resulting images (Figure 5a,d in main text) were smoothed with a gaussian filter (SD = 2).

Baseline pupil gain vs. loss

We confirmed that baseline pupil did not differ as a function of gain or loss condition. To do this, we coded each trial based on whether it was preceded by a loss trial or a gain trial. Using paired samples *t*-tests, the within-group median baseline pupil values did not differ based on whether the preceding trial was gain vs. loss [Atomoxetine: $t(18) = 1.51, p = 0.149$; Placebo: $t(18) = -0.40, p = 0.696$; Control: $t(24) = -0.54, p = 0.593$]. To determine whether the drug effect we saw on baseline pupil might depend on the preceding trial type we then performed a model comparison, similar to the model used to compare baseline pupil in the main manuscript (i.e., controlling for session order effects and repeated measures), but including the preceding trial type as an interaction term. For atomoxetine vs. placebo the interaction effect was not significant [$F(1, 18) = 3.69$, $p = 0.071$], nor was it significant in the placebo vs. control comparison [$F(1, 42) = 0.01$, $p = 0.906$]. Taken together, these results suggest that baseline pupil was not influenced by previous trial type, and further support the approach we took to collapse across gain and loss trials for the baseline pupil analysis.

Supplementary Figure 10

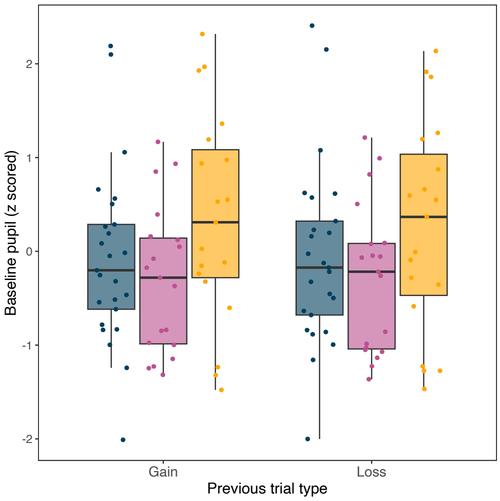

Supplementary Figure 10 – Median values from each for each subject from their pre-trial baselines, averaged across all trials and plotted as a function of whether a baseline was preceded by a gain or loss trial.

References

1. Gabry, J., Simpson, D., Vehtari, A., Betancourt, M. & Gelman, A. Visualization in Bayesian workflow. *Journal of the Royal Statistical Society: Series A (Statistics in Society)* **182**, 389–402 (2019).
2. Vehtari, A., Gelman, A. & Gabry, J. Practical Bayesian model evaluation using leave-one-out cross-validation and WAIC. *Stat Comput* **27**, 1413–1432 (2017).
3. Sivula, T., Magnusson, M. & Vehtari, A. Uncertainty in Bayesian Leave-One-Out Cross-Validation Based Model Comparison. *arXiv:2008.10296 [stat]* (2020).
4. Goetz, C. G. *et al.* Movement Disorder Society-sponsored revision of the Unified Parkinson's Disease Rating Scale (MDS-UPDRS): Scale presentation and clinimetric testing results. *Movement Disorders* **23**, 2129–2170 (2008).
5. Mioshi, E., Dawson, K., Mitchell, J., Arnold, R. & Hodges, J. R. The Addenbrooke's Cognitive Examination Revised (ACE-R): a brief cognitive test battery for dementia screening. *International Journal of Geriatric Psychiatry* **21**, 1078–1085 (2006).
6. Zigmond, A. S. & Snaith, R. P. The Hospital Anxiety and Depression Scale. *Acta Psychiatrica Scandinavica* **67**, 361–370 (1983).
7. Patton, J. H., Stanford, M. S. & Barratt, E. S. Factor structure of the barratt impulsiveness scale. *Journal of Clinical Psychology* **51**, 768–774 (1995).
8. Conners, C. K. *et al.* Self-ratings of ADHD symptoms in adults I: Factor structure and normative data. *J Atten Disord* **3**, 141–151 (1999).
9. Starkstein, S. E. *et al.* Reliability, validity, and clinical correlates of apathy in Parkinson's disease. *Journal of Neuropsychiatry and Clinical Neurosciences* 134 (1992).
10. Fehnel, S. E., Bann, C. M., Hogue, S. L., Kwong, W. J. & Mahajan, S. S. The development and psychometric evaluation of the motivation and energy inventory (MEI). *Qual Life Res* **13**, 1321–1336 (2004).
11. Stiasny-Kolster, K. *et al.* The REM sleep behavior disorder screening questionnaire—A new diagnostic instrument. *Movement Disorders* **22**, 2386–2393 (2007).
12. Wear, H. J. *et al.* The Cambridge Behavioural Inventory revised. *Dementia & Neuropsychologia* **2**, 102–107 (2008).
13. Kubinec, R. Ordered Beta Regression: A Parsimonious, Well-Fitting Model for Survey Sliders and Visual Analog Scales. *SocArXiv* (2020) doi:10.31235/osf.io/2sx6y.
14. Cohen, J. *Statistical Power Analysis for the Behavioral Sciences.* (Routledge, New York, 1988).
15. Kruschke, J. K. Rejecting or Accepting Parameter Values in Bayesian Estimation. *Advances in Methods and Practices in Psychological Science* **1**, 270–280 (2018).
16. Zhang, L., Lengersdorff, L., Mikus, N., Gläscher, J. & Lamm, C. Using reinforcement learning models in social neuroscience: frameworks, pitfalls and suggestions of best practices. *Social Cognitive and Affective Neuroscience* **15**, 695–707 (2020).
