# Supplementary material for: Pharmacological and pupillary evidence for the noradrenergic contribution to reinforcement learning in Parkinson’s disease
Source: Commun Biol. 2025 Aug 14;8:1223. doi: 10.1038/s42003-025-08627-2 (PMC12354755; doi:10.1038/s42003-025-08627-2)
Supplement: Supplementary file 2 — Reporting Summary [file 42003_2025_8627_MOESM2_ESM.pdf]

nature portfolio

Corresponding author(s): Claire O'Callaghan

Last updated by author(s): Jul 24, 2025

Reporting Summary

Nature Portfolio wishes to improve the reproducibility of the work that we publish. This form provides structure for consistency and transparency in reporting. For further information on Nature Portfolio policies, see our Editorial Policies and the Editorial Policy Checklist.

Statistics

For all statistical analyses, confirm that the following items are present in the figure legend, table legend, main text, or Methods section.

n/a	Confirmed	
☐	☒	The exact sample size (*n*) for each experimental group/condition, given as a discrete number and unit of measurement
☐	☒	A statement on whether measurements were taken from distinct samples or whether the same sample was measured repeatedly
☐	☒	The statistical test(s) used AND whether they are one- or two-sided<br>*Only common tests should be described solely by name; describe more complex techniques in the Methods section.*
☐	☒	A description of all covariates tested
☐	☒	A description of any assumptions or corrections, such as tests of normality and adjustment for multiple comparisons
☐	☒	A full description of the statistical parameters including central tendency (e.g. means) or other basic estimates (e.g. regression coefficient) AND variation (e.g. standard deviation) or associated estimates of uncertainty (e.g. confidence intervals)
☐	☒	For null hypothesis testing, the test statistic (e.g. *F*, *t*, *r*) with confidence intervals, effect sizes, degrees of freedom and *P* value noted<br>*Give P values as exact values whenever suitable.*
☐	☒	For Bayesian analysis, information on the choice of priors and Markov chain Monte Carlo settings
☐	☒	For hierarchical and complex designs, identification of the appropriate level for tests and full reporting of outcomes
☒	☐	Estimates of effect sizes (e.g. Cohen's *d*, Pearson's *r*), indicating how they were calculated

Our web collection on statistics for biologists contains articles on many of the points above.

Software and code

Policy information about availability of computer code

Data collection	Pupil data collected using Eyelink Portable Duo running a task that was programmed in Python (version 2.7).
Data analysis	Behavioural modelling was implemented in Stan and was run using the rstan interface package in R version 3.6.1. Analyses and visualisation conducted in R version 4.2.1, using open source packages: bayestestR , permutes, plausiblecor, afex, emmeans

For manuscripts utilizing custom algorithms or software that are central to the research but not yet described in published literature, software must be made available to editors and reviewers. We strongly encourage code deposition in a community repository (e.g. GitHub). See the Nature Portfolio guidelines for submitting code & software for further information.

Data

Policy information about availability of data

All manuscripts must include a data availability statement. This statement should provide the following information, where applicable:

- Accession codes, unique identifiers, or web links for publicly available datasets
- A description of any restrictions on data availability
- For clinical datasets or third party data, please ensure that the statement adheres to our policy

Data to reproduce manuscript figures and analysis are openly available to download here https://osf.io/7ez5r/.

Research involving human participants, their data, or biological material

Policy information about studies with human participants or human data. See also policy information about sex, gender (identity/presentation), and sexual orientation and race, ethnicity and racism.

Reporting on sex and gender	Sex distribution of groups is reported in Table one. Sex-based analyses were not conduted due to small sample size and uneven distribution of males:females
Reporting on race, ethnicity, or other socially relevant groupings	These data were not collected and are not reported
Population characteristics	Nineteen people with idiopathic Parkinson’s disease were recruited via the University of Cambridge Parkinson’s disease research clinic and the Parkinson’s UK volunteer network. All participants met the United Kingdom Parkinson’s Disease Society Brain Bank criteria, were aged between 50-80 years, with Hoehn and Yahr stages 1.5-3, and had no contraindications to 7T MRI or atomoxetine. No participant met clinical criteria for dementia or had an impulse control disorder. Twenty-six age-, sex- and education-matched healthy control participants were recruited via local volunteer panels. Control participants were screened for a history of psychiatric or neurological disorders, and were not taking psychoactive medications.
Recruitment	people with idiopathic Parkinson’s disease were recruited via the University of Cambridge Parkinson’s disease research clinic and the Parkinson’s UK volunteer network. healthy control participants were recruited via local volunteer panels
Ethics oversight	The study was approved by the Cambridge Research Ethics Committee (REC 10/H0308/34) and participants provided written informed consent. The study is registered on ISRCTN registry with study ID ISRCTN46299660 (https://doi.org/10.1186/ISRCTN46299660). The study was retrospectively registered because it was exempt from Clinical Trials status by the UK Medicines and Healthcare Products Regulatory Authority (MHRA)

Note that full information on the approval of the study protocol must also be provided in the manuscript.

Field-specific reporting

Please select the one below that is the best fit for your research. If you are not sure, read the appropriate sections before making your selection.

☒ Life sciences ☐ Behavioural & social sciences ☐ Ecological, evolutionary & environmental sciences

For a reference copy of the document with all sections, see nature.com/documents/nr-reporting-summary-flat.pdf

Life sciences study design

All studies must disclose on these points even when the disclosure is negative.

Sample size	Sample size was chosen based on the inclusion criteria for the study: no contraindications to 7T MRI or atomoxetine. This restrictive criteria limited us to n=19 people with Parkinson's disease. And the control sample was recruited to closely match that number. This sample size is consistent with other single-dose drug studies in Parkinson's and related conditions, and was deemed acceptable
Data exclusions	No exclusions
Replication	Replication was not done. This is a cross-over design.
Randomization	double-blind randomised placebo-controlled crossover design
Blinding	double-blind randomised placebo-controlled crossover design,

Reporting for specific materials, systems and methods

We require information from authors about some types of materials, experimental systems and methods used in many studies. Here, indicate whether each material, system or method listed is relevant to your study. If you are not sure if a list item applies to your research, read the appropriate section before selecting a response.

Materials & experimental systems

n/a	Involved in the study	
☒	☐	Antibodies
☒	☐	Eukaryotic cell lines
☒	☐	Palaeontology and archaeology
☒	☐	Animals and other organisms
☐	☒	Clinical data
☒	☐	Dual use research of concern
☒	☐	Plants

Methods

n/a	Involved in the study	
☒	☐	ChIP-seq
☒	☐	Flow cytometry
☒	☐	MRI-based neuroimaging

Clinical data

Policy information about clinical studies

All manuscripts should comply with the ICMJE guidelines for publication of clinical research and a completed CONSORT checklist must be included with all submissions.

Clinical trial registration: The study is registered on ISRCTN registry with study ID ISRCTN46299660 (https://doi.org/10.1186/ISRCTN46299660)

Study protocol: SRCTN registry with study ID ISRCTN46299660 (https://doi.org/10.1186/ISRCTN46299660)

Data collection: University of Cambridge 24/04/2017 to 15/04/2019

Outcomes: Primary outcome measure Measured 2 hours after taking drug/placebo, defined as changes in performance on cognitive tasks in the atomoxetine condition vs. on placebo

Plants

Seed stocks: *Report on the source of all seed stocks or other plant material used. If applicable, state the seed stock centre and catalogue number. If plant specimens were collected from the field, describe the collection location, date and sampling procedures.*

Novel plant genotypes: *Describe the methods by which all novel plant genotypes were produced. This includes those generated by transgenic approaches, gene editing, chemical/radiation-based mutagenesis and hybridization. For transgenic lines, describe the transformation method, the number of independent lines analyzed and the generation upon which experiments were performed. For gene-edited lines, describe the editor used, the endogenous sequence targeted for editing, the targeting guide RNA sequence (if applicable) and how the editor was applied.*

Authentication: *Describe any authentication procedures for each seed stock used or novel genotype generated. Describe any experiments used to assess the effect of a mutation and, where applicable, how potential secondary effects (e.g. second site T-DNA insertions, mosiacism, off-target gene editing) were examined.*
